# Off-centered Double-slit Metamaterial for Elastic Wave Polarization Anomaly

**DOI:** 10.1038/s41598-017-15746-2

**Published:** 2017-11-13

**Authors:** Hyung Jin Lee, Je-Ryung Lee, Seung Hwan Moon, Tae-Jin Je, Eun-chae Jeon, Kiyean Kim, Yoon Young Kim

**Affiliations:** 1 Institute of Advanced Machines and Design, Seoul National University, 1 Gwanak-ro, Gwanak-gu, Seoul, 08826 South Korea; 2 Korea Institute of Machinery and Materials (KIMM), 156 Gajeongbuk-ro, Yuseong-gu, Daejeon, 34103 South Korea; 3 School of Mechanical and Aerospace Engineering, Seoul National University, 1 Gwanak-ro, Gwanak-gu, Seoul, 08826 South Korea; 4 WCU Multiscale Mechanical Design Division, School of Mechanical and Aerospace Engineering, Seoul National University, 1 Gwanak-ro, Gwanak-gu, Seoul, 08826 South Korea

## Abstract

The polarization anomaly refers to the polarization transition from longitudinal to shear modes along an equi-frequency contour of the same branch, which occurs only in some anisotropic elastic media, but the lack of natural materials exhibiting desired anisotropy makes its utilization impossible for potential novel applications. In this paper, we present a unique, non-resonant type elastic metamaterial made of off-centered, double-slit unit cells. We show that its wave polarization characteristics that determine the desired anomalous polarization for a certain application are tailorable. As an application, a mode converting wedge that transforms pure longitudinal into pure shear modes is designed by the proposed metamaterial. The physics involved in the mode conversion is investigated by simulations and experiments.

## Introduction

Elastic waves possess distinct wave polarization characteristics that are different from electromagnetic and acoustic waves. The rich variety of polarizations, longitudinal and shear (transverse) in bulk media, and the strong coupling between the dissimilar modes are notable examples^1–3^. The unique, complex nature of elastic waves in polarization manifests some interesting wave phenomena that cannot be found in other wave fields. Among others, the “polarization anomaly” is a very unusual phenomenon^1,4–7^. It refers to the polarization transition from longitudinal to shear modes when the wavevector is moved along a certain equi-frequency contour (EFC) branch. Considerable research in the past few decades have paid attention to the theoretical demonstration on the phenomenon, but no actual applications have been explored thus far due to the lack of natural materials exhibiting the desired phenomenon.

Polarization is an important wave characteristic of vector waves like electromagnetic and elastic waves. In the electromagnetic field, the precise control of the wave polarization state is desired for numerous applications including optical communications, spectroscopy, microscopy, etc^8–12^. Birefringence, dichroism, or Brewster angle effect, among others, are commonly used to tailor the polarization-dependent dispersion property. For longitudinal and shear polarizations of elastic waves, similar phenomena may be employed to manipulate the relative dispersion property. Unlike two orthogonal transverse modes in electromagnetics, however, the dissimilar, longitudinal and shear, modes in elasticity involve far different physical behaviors. Such behaviors are dilatation (volume change) and dilatation-less (shape change) deformations where their relative properties are restrictively tailorable. This is because normal stiffness governing the longitudinal mode is larger than shear stiffness governing the shear mode for usual solids even with strong anisotropy. Equivalently, it means that the (quasi-)longitudinal velocity is faster than (quasi-)shear velocity for usual solids.

To the best of our knowledge, the polarization anomaly is the one and only phenomenon that breaks the usual dispersion relation between the in-plane longitudinal and shear modes without any resonance scheme. The relative dispersion–not only frequency-dependent dispersion, but also spatial dispersion–properties can be reversed between the modes when we use the anomaly. Thus, the phenomenon has great potential for a variety of applications like retardation, filtering, controlling of scattering and radiation, tailoring of polarization state, and so on. Unfortunately, the artificial realization of the anomaly in the composite form is quite difficult due to the peculiar anisotropic material property; it requires smaller normal stiffness than shear stiffness for specific material axes while normal stiffness should be larger than shear stiffness for other material axes^5–7^. Due to the classic paradigms for composite design, the required material property is not achievable as long as one uses natural ingredients^5,6,13^.

In this paper, we first propose an elastic metamaterial the rectangular unit cell of which consists of off-centered double slits. The novelty of our metamaterial resides in the special “off-centered” slit arrangement, critical for the realization of polarization anomaly. In this arrangement, the slit length in particular has a significant effect on the relative ratio of effective shear stiffness to effective normal stiffness for the perpendicular material axis to the slits. Therefore, an extremely wide range of stiffness control, even covering the peculiar property of smaller normal stiffness than shear stiffness, is found achievable. We consider a non-resonant type metamaterial here since its working frequency range is considerably broader than that by resonant type metamaterials, ensuring stability and robustness in performance. Some simulations were implemented to show how the slit dimensions affect the effective characteristics of the metamaterial and experiments with the metamaterial-based mode-converting wedge were performed to verify the realization of polarization anomaly for the desired wave manipulation.

## Results

### Polarization anomaly

The unusual polarization transition from longitudinal to shear modes within a single EFC is called “polarization anomaly” or “anomalous polarization”^4,5^. This phenomenon is known to occur in a rare anisotropic solid such as a transversely isotropic or orthorhombic symmetry of solid. Calcium formate, which is orthorhombic, is one of the known crystals that exhibit the anomaly. Unlike other usual solids, calcium formate possesses peculiar material properties such that normal stiffness is larger than shear stiffness for a specific material axis while shear stiffness is larger than normal stiffness for an axis orthogonal to the axis. This abnormal behavior causes anomalous polarization.

**Figure 1:**
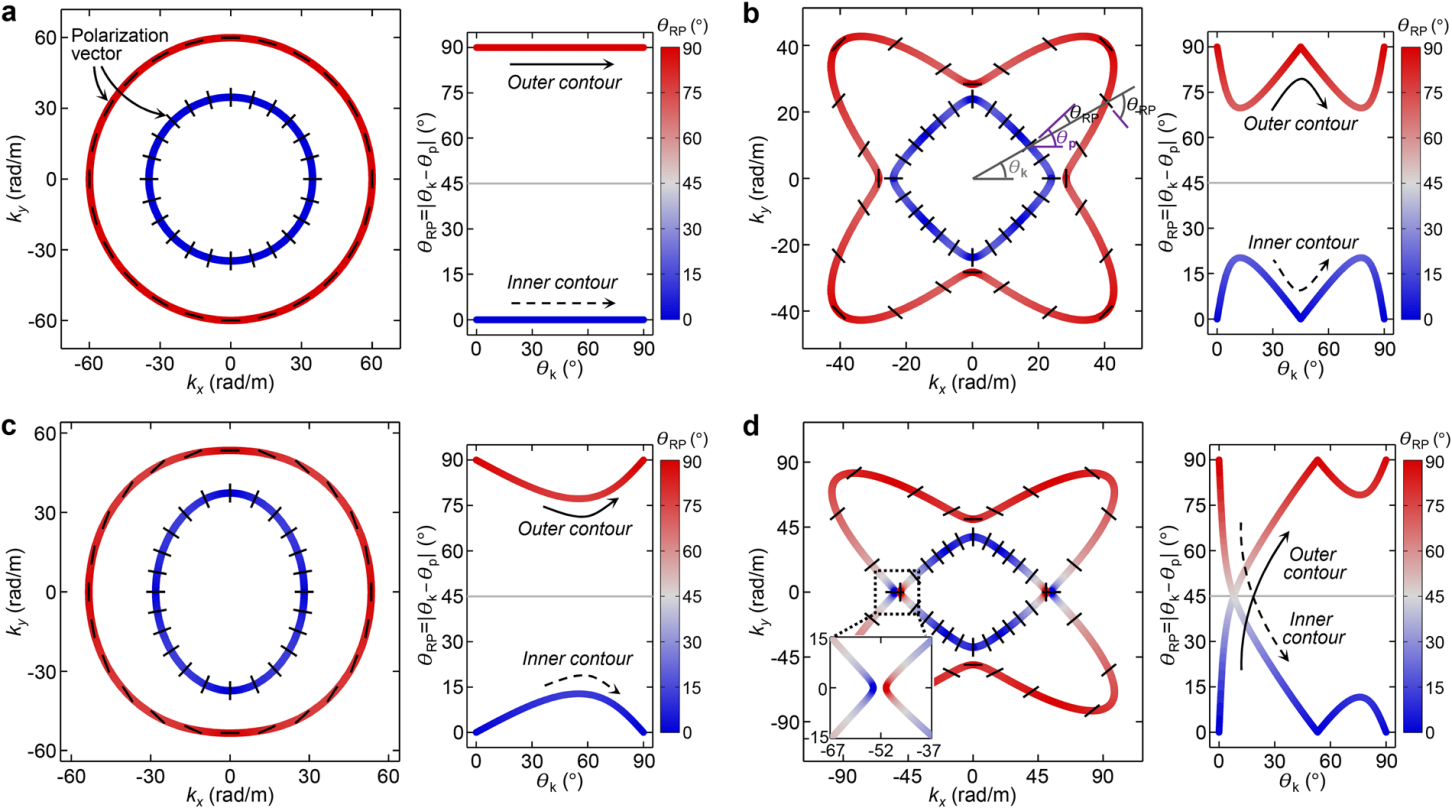
Equi-frequency contours (EFCs) and polarization characteristics of isotropic and anisotropic solids. The EFCs of in-plane wave modes (left) and their polarization orientations relative to the wavevector orientations (right) defined as ${\theta }_{{\rm{RP}}}=|{\theta }_{{\rm{p}}}-{\theta }_{{\rm{k}}}|$ for ${0}^{\circ }\le {\theta }_{{\rm{k}}}\le {90}^{\circ }$ (${\theta }_{{\rm{p}}}$: polarization orientation, ${\theta }_{{\rm{k}}}$: wavevector orientation) at 30 kHz. As an isotropic solid, (**a**) aluminum with anisotropic solids, (**b**) rutile, (**c**) barium sodium niobate, and (**d**) calcium formate are considered. When calculating ${\theta }_{{\rm{RP}}}$, the polarization orientation ${\theta }_{{\rm{p}}}$ that leads to ${0}^{\circ }\le {\theta }_{{\rm{RP}}}\le {90}^{\circ }$ is selected. The line color of the EFC and ${\theta }_{{\rm{RP}}}$ plots represent the value of ${\theta }_{{\rm{RP}}}$. Blue represents ${\theta }_{{\rm{RP}}}={0}^{\circ }$ (purely longitudinal) and red represents ${\theta }_{{\rm{RP}}}={90}^{\circ }$ (purely shear). In the EFC plots, the polarization vectors at a certain interval of ${\theta }_{{\rm{k}}}$ are also illustrated with black solid poles.

In order to explain the polarization anomaly phenomenon better, the EFCs and polarization characteristics of calcium formate are examined in Fig. 1d. They are compared with those of other ordinary solids like aluminum, rutile, and barium sodium niobate shown in Fig. 1a–c, respectively. The material properties are listed in Table 1. To facilitate analysis, only the in-plane (quasi-)longitudinal and (quasi-)shear modes in a two-dimensional space are considered. The polarization characteristics of the materials are evaluated by ${\theta }_{{\rm{RP}}}=|{\theta }_{{\rm{p}}}-{\theta }_{{\rm{k}}}|$ $({0}^{\circ }\le {\theta }_{{\rm{RP}}}\le {90}^{\circ })$, which denotes the relative angle of the polarization orientation $({\theta }_{{\rm{p}}})$ with respect to the wavevector orientation $({\theta }_{{\rm{k}}})$. The result is also reflected in the EFC and *θ*_RP_ plots by the line color where blue represents *θ*_RP_ = 0° (purely longitudinal) and red represents *θ*_RP_ = 90° (purely shear). In the EFC plots, the polarization vectors are also illustrated with the black solid poles at the wavevectors with an interval of *θ*_k_ = 15°.

**Table 1:** Material properties (mass density *ρ*, stiffness components *C*_11_, *C*_12_, *C*_22_, and *C*_66_) of aluminum, rutile, barium sodium niobate, and calcium formate.

Material	*ρ* (kg/m^3^)	*C*_11_ (10^10^ Pa)	*C*_12_ (10^10^ Pa)	*C*_22_ (10^10^ Pa)	*C*_66_ (10^10^ Pa)
Aluminum	2700	7.97	2.63	7.97	2.67
Rutile	4260	26.60	17.33	26.60	18.86
Barium sodium niobate	5300	23.90	5.00	13.50	6.60
Calcium formate	2020	2.44	2.48	4.92	2.82

By comparing Fig. 1d for calcium formate and Fig. 1a–c for ordinary solids, one can see a clear distinction in their polarization characteristics; in the outer (inner) EFC branch of calcium formate, polarization is changed from being purely longitudinal (*θ*_RP_ = 0°) to being purely shear (*θ*_RP_ = 90°) (purely shear to purely longitudinal for the inner branch) as *θ*_k_ is moved from 0° to 90°. No such behavior appears in the EFCs of other ordinary solids because they exhibit monotonic polarization behaviors such that (quasi-)longitudinal and (quasi-)shear polarizations always remain in the inner and outer branches, respectively. In these materials, *θ*_RP_ varies only between 0° and 45° for one EFC branch and between 45° and 90° for the other branch. The amount of the *θ*_RP_ variation can differ depending on material anisotropy, but this observation remains valid for ordinary materials. (See Supplementary A for more details.) This means that the usual dispersion property of fast (quasi-)longitudinal and slow (quasi-)shear modes is observed for ordinary solids. For calcium formate, on the other hand, the (quasi-)shear wave velocity is observed to exceed the (quasi-)longitudinal wave velocity at around *θ*_k_ = 0° and 180°, due to the anomalous polarization transition in the EFCs. The polarization anomaly originates from the peculiar material anisotropy such that *C*_11_ < *C*_66_ < *C*_22_^6^. Here, *C*_11_ and *C*_22_ denote the normal stiffnesses for the *x*- and *y*-directions and *C*_66_, the shear stiffness in the *x*-*y* plane. As a matter of fact, the condition that *C*_11_ < *C*_66_ < *C*_22_ always guarantees the polarization anomaly regardless of the values of *C*_12_. (See Supplementary B for more details.) In ordinary solids, such anisotropy is hard to find since normal stiffness is supposed to be larger than shear stiffness^1,5,7^.

### Metamaterial design

The key to realizing the polarization anomaly is in (i) effectively achieving very low normal stiffness even below shear stiffness for a specific material axis while (ii) keeping the usual constitutive relation of higher normal stiffness than shear stiffness for other material axes. More importantly, the constitutive parameters responsible for the anomaly should be tailorable for a wide frequency range to guarantee stable and robust performances as desired for practical applications. It is definitely challenging to fulfill these requirements by employing a composite, especially if one does not use any locally resonant element. Even with a resonance scheme, the elaborate control of required anisotropy in a certain frequency range is highly questionable.

**Figure 2:**
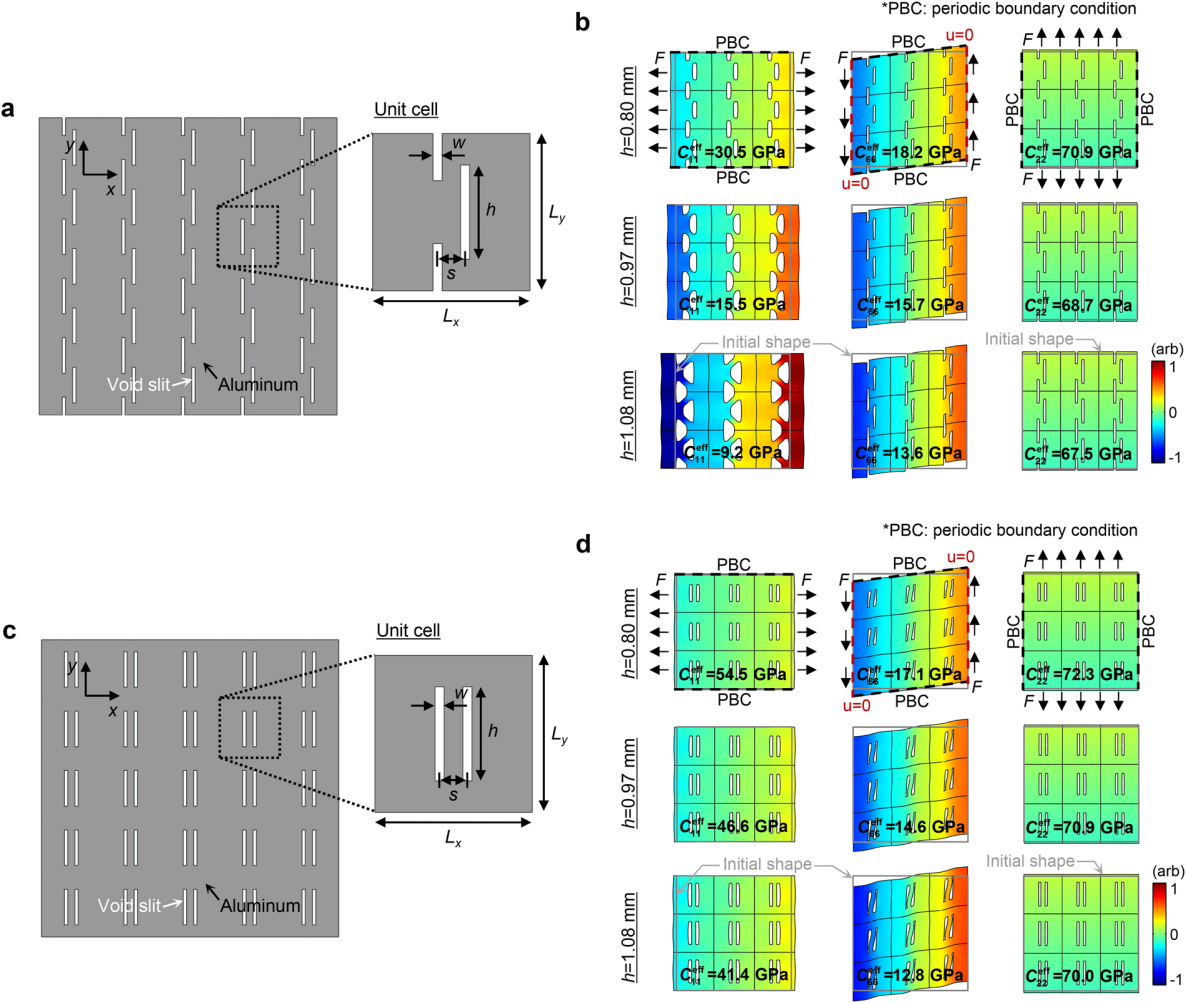
Off-centered double-slit metamaterial and centered double-slit metamaterial. (**a**) The schematic configuration of the proposed off-centered double-slit metamaterial and (**b**) the static deforming behaviors under the *x*-directional dilatational load (first column), shear load (second), and *y*-directional dilatational load (third). The surface colors for the plots in the first, second and third columns represent the *x*-, *y*-, and *y*-directional displacements. Three different slit lengths, $h=0.80$ (first row), 0.97 (second), and 1.08 mm (third), are considered in the simulations. The same magnitude of force (*F*) is applied at the outer metamaterial boundaries, and the periodic boundary condition is imposed on the other boundaries (marked with the black dashed lines). For the pure shear deformation, the constraint that makes no *x*-directional displacement is used for the left and right boundaries (marked with red dashed lines). The effective stiffnesses ${C}_{11}^{{\rm{eff}}}$, ${C}_{66}^{{\rm{eff}}}$, and ${C}_{22}^{{\rm{eff}}}$ involved in each deformation are calculated by the stress-strain relations. (For instance, ${C}_{11}^{{\rm{eff}}}$ is defined as ${\sigma }_{xx}/{\epsilon }_{xx}=\{F/(3{L}_{y}t)\}/\{{\delta }_{x}/(3{L}_{x})\}$ where *t* is the thickness of the unit cell in the out-of-plane direction and ${\delta }_{x}$ is the relative horizontal displacement between the two force-acting surfaces.) In every plot, the deformations are somewhat exaggerated for clarity. (**c**) For reference, the configuration of the centered double-slit metamaterial is also considered and (**d**) same static analyses are conducted for the metamaterial.

In Fig. 2a, we propose a specially-configured elastic metamaterial in order to realize the polarization anomaly. The rectangular unit cell consists of off-centered double slits. As demonstrated later, the special arrangement of the slits not producing any local resonance has a pivotal role in achieving the desired anisotropy for the polarization anomaly. The metamaterial is considered two-dimensional under the plane stress condition^14^ and aluminum (mass density ${\rho }_{0}=2700\,{\mathrm{kg}/m}^{3}$, Young’s modulus ${E}_{0}=71\,{\rm{GPa}}$, Poisson’s ratio ${\nu }_{0}=0.33$) is used as the substrate. To show that the effective wave property of the metamaterial can be elaborately engineered, the slit length parameter *h*, one of the crucial parameters determining the metamaterial behavior, is varied while the other geometric parameters are fixed. The lattice constants for the *x*- and *y*-directions are the same as $L={L}_{x}={L}_{x}=1.8\,{\rm{mm}}$ while the slit width is $w=0.1\,{\rm{mm}}$ and the separation distance between the adjacent slits, $s=0.3\,{\rm{mm}}$ (other varying parameters influencing the effective behavior are investigated in Supplementary C).

Some static analyses are conducted in Fig. 2b to determine the effective deforming behavior of the proposed metamaterial. The dilatational and shear deformations in the principal axes are specifically evaluated (detailed simulation setups are illustrated in the first-row plots), and the effective stiffnesses involved in the deformations, the effective normal stiffnesses ${C}_{11}^{{\rm{eff}}}$ and ${C}_{22}^{{\rm{eff}}}$ for the *x*- and *y*-directions, respectively, and the effective shear stiffness ${C}_{66}^{{\rm{eff}}}$ are calculated from the stress-strain relation. Note that the effective stiffnesses derived here are static and frequency-independent, indicating that the stiffnesses have nothing to do with resonance. In the simulations, three different slit lengths–*h* = 0.80 (the first row), 0.97 (second), and 1.08 mm (third)–are considered. In order to show the uniqueness of the special off-centered slit arrangements in the proposed metamaterial, the effective behavior is compared to the metamaterial with an array of conventional double slits (see Fig. 2c,d where the same geometric parameters are considered under the same simulation setups as in Fig. 2a,b).

One clear distinction exists for effective behavior of the two different metamaterial models. In particular, for the dilatational deformation in the *x*-direction, the proposed off-centered double-slit metamaterial is sensitive to the increase in slit length *h* while the conventional double-slit metamaterial is not. For the shear and *y*-directional dilatational deformations, on the other hand, a quite similar behavior is found between the models. Furthermore, they are not altered much by the increase in *h*. The observations can be confirmed by the retrieved effective constitutive parameters. In the proposed off-centered double-slit model, the effective normal stiffness ${C}_{11}^{{\rm{eff}}}$, as opposed to ${C}_{66}^{{\rm{eff}}}$ and ${C}_{22}^{{\rm{eff}}}$, dramatically decreases as *h* increases. Under the off-centered arrangement, the desired constitutive relationship for the polarization anomaly is achieved with *h* = 1.08 mm, resulting in the relation that ${C}_{11}^{{\rm{eff}}} < {C}_{66}^{{\rm{eff}}} < {C}_{22}^{{\rm{eff}}}$. In the conventional double-slit model, the extraordinary flexibility for the *x*-directional dilatation involved with ${C}_{11}^{{\rm{eff}}}$ is not observed for any *h*. The extraordinary macroscopic flexibility mainly comes from the significant reduction in the local stiffness particularly at the region surrounded by the adjacent slits. The unique off-centered slit arrangement makes the structural component at the center of unit cell exhibits bending-dominant motion for a dilatational load the corresponding stiffness of which is much weaker than usual dilatational stiffness. (See Supplementary D for more details.) The argument can be confirmed by the abrupt change of displacement field (represented by surface color) at these areas, which implies large strain. Due to the special slit arrangements, the desired peculiar material anisotropy is achieved with a single-phase common material like aluminum. No local resonance or special ingredients are used.

### Effective wave behavior

**Figure 3:**
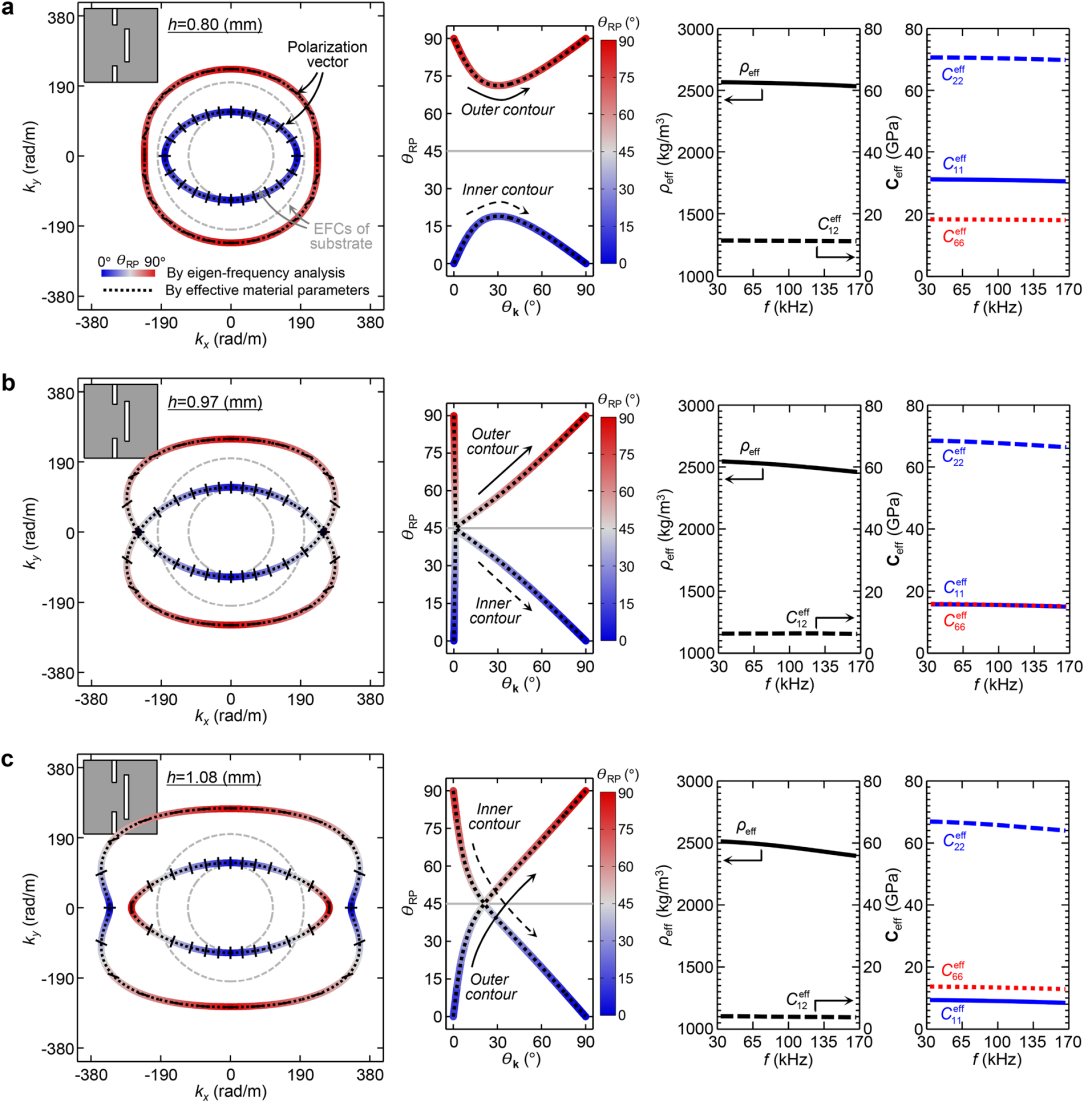
Effective wave and material properties of the metamaterial for varying slit lengths. The EFCs of in-plane wave modes (left) and their relative polarization orientation to the wavevector orientation ${\theta }_{{\rm{RP}}}$ for ${0}^{\circ }\le {\theta }_{{\rm{k}}}\le {90}^{\circ }$ (middle) of the proposed off-centered double-slit metamaterial at $f=100\,{\rm{kHz}}$. The effective scalar mass density ${\rho }_{{\rm{eff}}}$ and the effective stiffnesses ${C}_{11}^{{\rm{eff}}}$, ${C}_{12}^{{\rm{eff}}}$, ${C}_{22}^{{\rm{eff}}}$, and ${C}_{66}^{{\rm{eff}}}$ are retrieved for the frequency range of $30\,{\rm{kHz}}\le f\le 170\,{\rm{kHz}}$ (right). The analyses are conducted for three different slit lengths: (**a**) $h=0.80$, (**b**) 0.97, and (**c**) 1.08 mm. The EFCs and ${\theta }_{{\rm{RP}}}$ are calculated by using two different methods: one from the eigen-frequency analysis (indicated by the color representing the value of ${\theta }_{{\rm{RP}}}$) and the other from the retrieved effective material parameters (indicated by black dotted lines). In the EFC plots, the polarization vectors at a certain interval of ${\theta }_{{\rm{k}}}$ are marked by black solid poles. In addition, the EFCs of aluminum substrate are indicated by gray dashed lines.

In order to demonstrate the actual wave (dynamic) behavior of the proposed metamaterial, the EFCs and polarization characteristics are examined in Fig. 3 by employing the eigen-frequency analysis. Three different slit lengths–*h* = 0.80, 0.97, and 1.08 mm–are considered here again. The analyses are conducted at $f=100\,{\rm{kHz}}$ where the typical long-wavelength condition is satisfied. To evaluate the polarization characteristics, the relative polarization orientation to the wavevector orientation (*θ*_RP_) is calculated just as in Fig. 1. The results are also reflected by the line color in the EFC and *θ*_RP_ plots in Fig. 3. (See also the black solid poles in the EFC plots indicating the polarization vectors.) Furthermore, the total effective material parameters with the effective scalar mass density ${\rho }_{{\rm{eff}}}$ and the effective stiffnesses for ${C}_{11}^{{\rm{eff}}}$, ${C}_{12}^{{\rm{eff}}}$, ${C}_{22}^{{\rm{eff}}}$, and ${C}_{66}^{{\rm{eff}}}$ are retrieved for the $30\,{\rm{kHz}}\le f\le 170\,{\rm{kHz}}$ frequency range by using the S-parameter retrieval method for anisotropic elastic metamaterials^15^. The EFCs and *θ*_RP_ are also calculated from the retrieved effective parameters (indicated with black dotted lines) to validate the results. The effective parameter accuracy is checked in terms of the scattering parameter in Supplementary E.

From the retrieved effective material parameters, the proposed metamaterial acquires fairly stable behavior throughout the entire low frequency range of interest regardless of *h*. Even though the effective parameters are a little dispersive along the frequency, probably due to the multiple scattering effect^15^, the effective stiffnesses ${C}_{11}^{{\rm{eff}}}$, ${C}_{22}^{{\rm{eff}}}$, and ${C}_{66}^{{\rm{eff}}}$, are well matched with those from the previous static analysis. Just as in the static results, the effective normal stiffness ${C}_{11}^{{\rm{eff}}}$ sensitively decreases to the increasing *h* while the other stiffnesses do not. For $h=1.08\,{\rm{mm}}$, the targeted peculiar anisotropic material relation of ${C}_{11}^{{\rm{eff}}} < {C}_{66}^{{\rm{eff}}} < {C}_{22}^{{\rm{eff}}}$ is consistently achieved in the broad low frequency regime.

Various classes of the effective material anisotropies of the metamaterial, depending on *h*, bring distinctively different wave behaviors for each case. When $h=0.80\,{\rm{mm}}$ as in Fig. 3a, the usual dispersion relation between the (quasi-)longitudinal and (quasi-)shear modes are found at any wavevector orientation. The (quasi-)longitudinal mode is faster than the (quasi-)shear mode, or equivalently the polarization in the fast branch is (quasi-)longitudinal while that in the slow branch is (quasi-)shear. On the other hand, when $h=0.97\,{\rm{mm}}$ as in Fig. 3b, singular dispersion and polarization behaviors are found. At ${k}_{y}=0$, the two EFC branches virtually meet through the cone-shaped curve vertices and the polarization states in the two branches drastically move to the transition point (*θ*_RP_ = 45°) in the vicinities of the singularities. A similar phenomenon with the conical singularity^16,17^ is obtained in the case due to the virtual equality between the effective normal stiffness and the effective shear stiffness (i.e., ${C}_{66}^{{\rm{eff}}}\approx {C}_{11}^{{\rm{eff}}}$). As *θ*_k_ moves away from the singularities, the usual polarization behavior is observed in both branches. When $h=1.08\,{\rm{mm}}$ as in Fig. 3c, the anomalous polarization transitions from longitudinal to shear (shear to longitudinal) are found in each EFC branch. The relative dispersion property between the (quasi-)longitudinal and (quasi-)shear modes for $0\le {\theta }_{{\rm{k}}} < {22}^{\circ }$ is in marked contrast to that for $22 < {\theta }_{{\rm{k}}}\le {90}^{\circ }$ where the polarization transitions (*θ*_RP_ = 45°) take place at ${\theta }_{{\rm{k}}}\approx {22}^{\circ }$. The polarization anomaly is more evidently achieved here than in calcium formate (Fig. 1d) by creating a broader anomaly region as well as higher contrast in the relative dispersion property between the longitudinal and shear modes, which may be more suitable for actual applications. Virtually the same dispersion relation and polarization characteristics are consistently obtained in the entire low frequency regime due to the stable effective behavior of the metamaterial.

**Figure 4:**
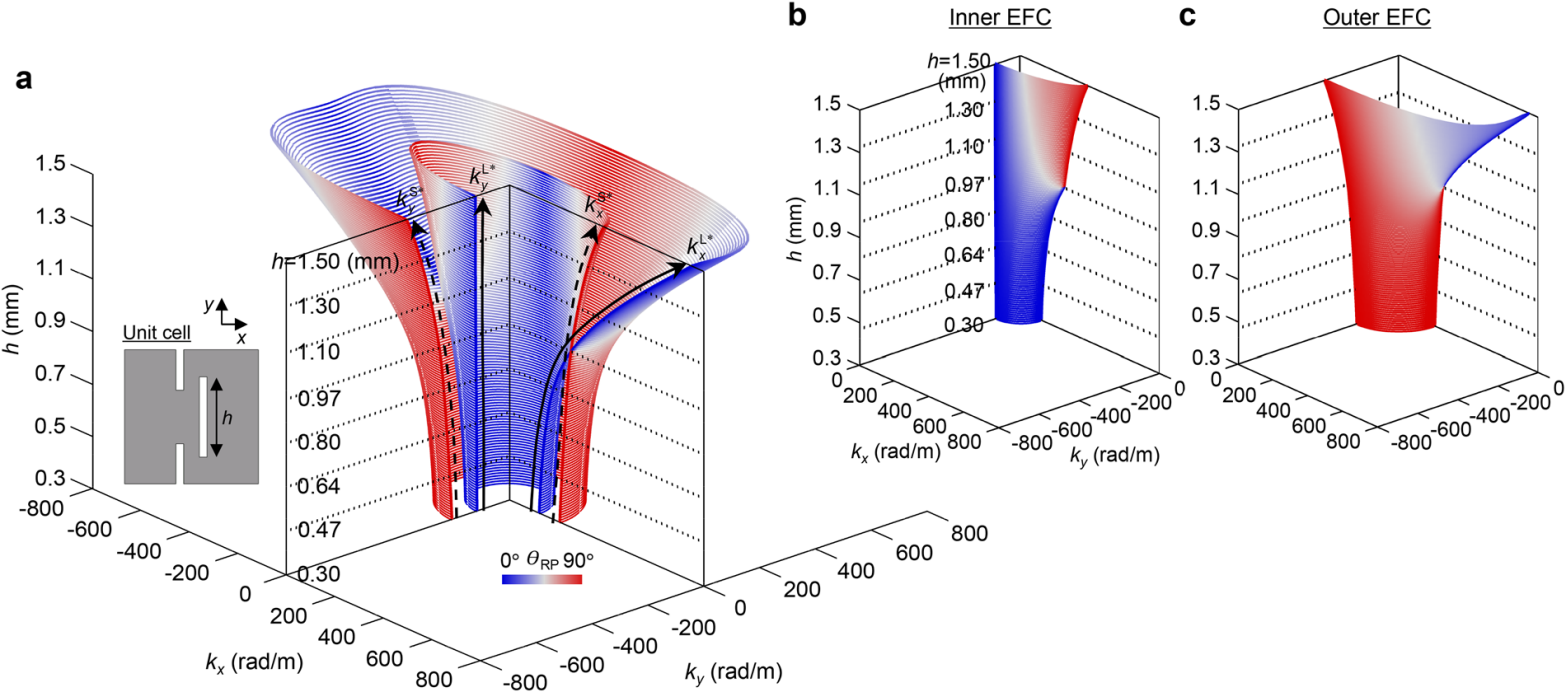
Variations in EFCs and polarization characteristics depending on the slit length of the metamaterial. The EFCs of in-plane wave modes in the proposed metamaterial are investigated for varying slit lengths between $0.3\,{\rm{mm}}\le h\le 1.5\,{\rm{mm}}$. The relative polarization orientation to the wavevector orientation ${\theta }_{{\rm{RP}}}$ is reflected by the line color. The EFCs are shown in all but the fourth quadrant region in **a**. The variations of the longitudinal and shear wavenumbers for the *x*-direction, ${k}_{x}^{{{\rm{L}}}^{\ast }}$ and ${k}_{x}^{{{\rm{S}}}^{\ast }}$, and those for the *y*-direction, ${k}_{y}^{{{\rm{L}}}^{\ast }}$ and ${k}_{y}^{{{\rm{S}}}^{\ast }}$, are illustrated by black solid and dashed lines. In **b** and **c**, the inner and outer EFC branches are shown separately for the fourth quadrant.

The overall dispersion and polarization characteristics of the off-centered double-slit metamaterial can be seen in Fig. 4 for varying slit lengths between $0.30\le h\le 1.50\,{\rm{mm}}$ derived using the eigen-frequency analysis at $f=100\,{\rm{kHz}}$. In Fig. 4a, the EFC profiles the line color of which represents the relative polarization orientation (*θ*_RP_) are shown except for the fourth quadrant region while the inner and outer EFCs are separately shown for the quadrant in Fig. 4b,c, respectively.

There are wavenumber variations along *h*, specifically when ${k}_{y}=0$ and ${k}_{x}=0$. For convenience, the corresponding longitudinal and shear wavenumbers are defined as ${k}_{x}^{{{\rm{L}}}^{\ast }}$ and ${k}_{x}^{{{\rm{S}}}^{\ast }}$, respectively, for ${k}_{y}=0$ as well as ${k}_{y}^{{{\rm{L}}}^{\ast }}$ and ${k}_{y}^{{{\rm{S}}}^{\ast }}$, respectively, for ${k}_{x}=0$. Their variations are highlighted with the black solid and dashed lines, respectively, in Fig. 4a. When considering ${k}_{x}^{{{\rm{L}}}^{\ast }}=\omega \sqrt{{\rho }_{{\rm{eff}}}/{C}_{11}^{{\rm{eff}}}}$, ${k}_{x}^{{{\rm{S}}}^{\ast }}={k}_{y}^{{{\rm{S}}}^{\ast }}=\omega \sqrt{{\rho }_{{\rm{eff}}}/{C}_{66}^{{\rm{eff}}}}$, and ${k}_{y}^{{{\rm{L}}}^{\ast }}=\omega \sqrt{{\rho }_{{\rm{eff}}}/{C}_{22}^{{\rm{eff}}}}$ where $\omega$ is the angular frequency, the wavenumber variations along *h* follow the exact variations of the involved stiffnesses, as observed in Figs 2 and 3. A remarkable variation can be found for ${k}_{x}^{{{\rm{L}}}^{\ast }}$ with relatively little variation for others. Due to the high contrast between the varying sensitivities of the wavenumbers for the ${k}_{y}=0$ direction, the usual dispersion relation between the longitudinal and shear modes (i.e., ${k}_{x}^{{{\rm{L}}}^{\ast }} < {k}_{x}^{{{\rm{S}}}^{\ast }}$) observed for $h < 0.97\,{\rm{mm}}$ are surprisingly reversed as ${k}_{x}^{{{\rm{S}}}^{\ast }} < {k}_{x}^{{{\rm{L}}}^{\ast }}$ for $h > 0.97\,{\rm{mm}}$. In the orthogonal ${k}_{x}=0$ direction, both the longitudinal and shear wavenumbers retain the usual dispersion relation as ${k}_{y}^{{{\rm{L}}}^{\ast }} < {k}_{y}^{{{\rm{S}}}^{\ast }}$ regardless of *h*. As evidently seen in Fig. 4b,c, the larger the slit length *h*, the broader the polarization anomaly region and the higher dispersion contrast between the slow and fast modes. Remarkably, the variations are gradual and the observations confirm that the dispersion property of the proposed metamaterial can be elaborately engineered just by tailoring the geometric slit parameters, especially slit length *h*. The non-resonant physics of the proposed metamaterial guarantees outstanding robustness for perturbations in the geometric parameters as well as other working environments like frequency.

### Numerical validations

**Figure 5:**
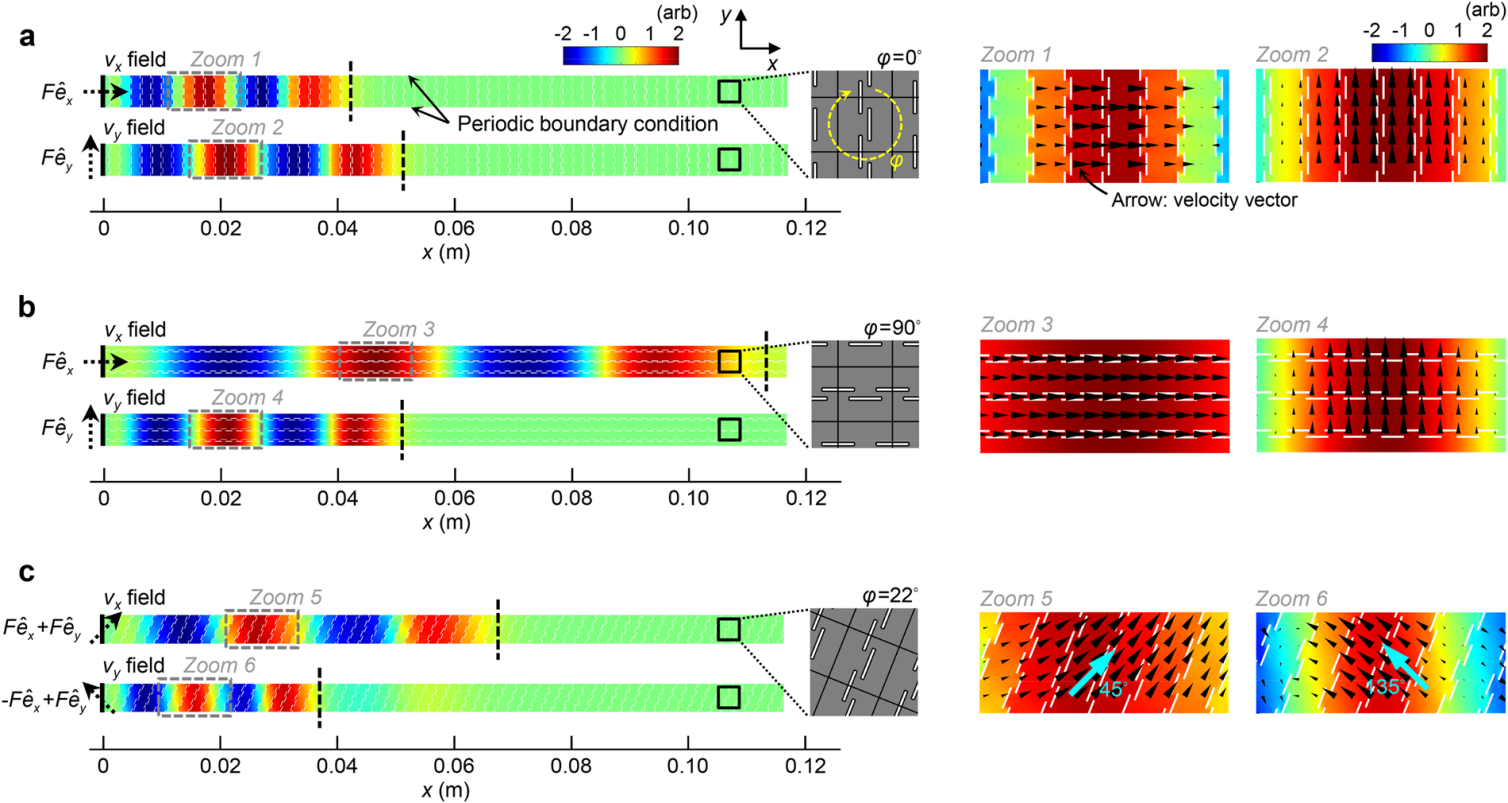
Time-transient simulations on the plane wave propagations in the metamaterial. The wave propagations at the wavevector orientations of (**a**) ${\theta }_{{\rm{k}}}={0}^{\circ }$, (**b**) 90°, and (**c**) 22° for the original metamaterial coordinates are considered. In each simulation, the wave propagation orientation is adjusted to be along the *x*-direction and the metamaterial coordinate is rotated by $\phi ={0}^{\circ }$, 90°, and 22°. As an input signal, a 100-kHz-sinusoidal wave with two cycles is excited at $x=0$. The detailed force vectors used for the wave excitations are illustrated beside the left boundary of the analysis domain where *F* indicates the arbitrary force amplitude. The symbols ${\hat{e}}_{x}$ and ${\hat{e}}_{y}$ denote the unit *x*- and *y*-directional base vectors, respectively. For the upper and lower boundaries, the periodic boundary condition is employed. The *x*- or *y*-directional velocity (${v}_{x}$ or ${v}_{y}$) field is captured at $t={t}^{\ast }=22\,\mu {\rm{s}}$. The leading wave fronts, calculated by using the effective material parameters of the metamaterial, are illustrated by black dashed lines. In the zoomed views (right) shown in Zooms 1–6, the velocity vectors (polarization vectors) are represented by black arrows.

In Fig. 5, we numerically examine the actual wave propagations in the proposed metamaterial that exhibit the polarization anomaly. The metamaterial with the slit length $h=1.08\,{\rm{mm}}$ the effective behavior of which is shown in Fig. 3c is adopted for the simulations. The plane wave propagations for the two orthogonal principal axes corresponding to the wavevector orientations of *θ*_k_ = 0° and 90° as well as the propagation for *θ*_k_ = 22° are considered in Fig. 5a–c. The direction of *θ*_k_ = 22° corresponds to the orientation where the polarization transition (*θ*_RP_ = 45°) takes place. The wave propagation orientations for each case are all adjusted to be along the *x*-direction while the metamaterial geometry is rotated by $\phi ={0}^{\circ }$, 90°, and 22° (the simulation method for *θ*_k_ = 22° is described in Supplementary F). At *x* = 0 in the left boundary, two sinusoidal force cycles are excited at the center frequency of $f=100\,{\rm{kHz}}$. Note that the force vectors (illustrated by the black dotted arrows) used for each case are set to match the polarization orientations of the wave modes that are targeted to generate. The time-transient analyses were implemented and the results were captured at a specific time ($t={t}^{\ast }=22\,\mu {\rm{s}}$) represented by the *x*- or *y*-directional velocity (${v}_{x}$ or ${v}_{y}$) field.

Let us first demonstrate the results for the propagations in the two principal axes, which correspond to $\phi ={0}^{\circ }$ (Fig. 5a) and $\phi ={90}^{\circ }$ (Fig. 5b). In terms of the relative dispersion property between the longitudinal (corresponding to the upper plots) and shear (lower plots) modes, disparate observations can be made for each case. (See zoomed views marked by Zooms 1–4 to evaluate the wave polarizations where the velocity vectors (polarization vectors) were illustrated with the black arrows.) For $\phi ={0}^{\circ }$, the shear wave unusually propagates faster than the longitudinal wave while being slower for $\phi ={90}^{\circ }$. Obviously, the extraordinary dispersion relation between the dissimilar wave modes is found for $\phi ={0}^{\circ }$. The observations confirm that the polarization anomaly occurs in the metamaterial. For $\phi ={22}^{\circ }$ in Fig. 5c, the fast (corresponding to the upper plot) and slow (lower plot) wave modes are generated. The polarization orientations of the wave modes are *θ*_p_=45° (−135°) and 135° (−45°) for the wavevector orientation of *θ*_k_ = 0°. They correspond to the relative polarization orientation of ${\theta }_{{\rm{RP}}}=|{\theta }_{{\rm{k}}}-{\theta }_{{\rm{p}}}|={45}^{\circ }$ where ${0}^{\circ }\le {\theta }_{{\rm{RP}}}\le {90}^{\circ }$. Remarkably, the wave modes are neither quasi-longitudinal nor quasi-shear.

Through theoretical and numerical analyses, we validated that the anomalous polarization phenomenon brings extraordinary wave propagations in terms of the frequency-dependent dispersion property as well as polarization property. For spatial dispersion property, one can also achieve interesting wave behavior from the anomaly by placing the (quasi-)longitudinal mode at the slow branch with the (quasi-)shear mode at the fast branch, as opposed to the usual mode locations in the wavevector space. In elasticity, the degree of freedom that can be obtained in the EFC curvature of the fast mode (corresponding to the inner EFC) is distinctively different from that of the slow mode (the outer EFC) in addition to the different anisotropy for the modes^16,18,19^. (See Supplementary G for more details.) The unique wave properties of the proposed metamaterial open up unprecedented opportunities to manipulate elastic waves for novel applications.

### Wave mode converting wedge

As a novel application of the polarization anomaly, we will create a wave mode converting wedge that can transform an incident pure longitudinal wave into a pure shear wave. In the engineering view point, this longitudinal-to-shear converter has critical applications for nondestructive evaluation, sound/vibration reduction, medical diagnosis, and more^20,21^. Especially in the nondestructive evaluation area, converting wedges have been by far most commonly utilized for angle beam inspection^2^. Almost all the conventional converters are built based on the critical angle scheme^2,22–24^, but they usually suffer from issues like low transduction efficiency and performance instability. Several recent studies^20,21,25^ have paid attention to metamaterials to develop a new type of converter, but pure conversion between the dissimilar elastic wave modes still remains a challenge.

Our main strategy for designing a wave mode converter is to implement the drastic wavevector change in the proposed metamaterial by using wave refraction. Specifically, by changing the wavevector orientation from *θ*_k_ = 0° to 90° while keeping the wavevector lying on the outer EFC branch (see EFCs in Fig. 3c), we may accomplish pure conversion from longitudinal to shear mode due to the polarization anomaly of the metamaterial. Remarkably, the conversion can take place thoroughly inside the metamaterial and not at the interfaces between the metamaterial and surrounding media as in the conventional critical-angle type. Below, our novel converting methodology will be demonstrated in more detail.

**Figure 6:**
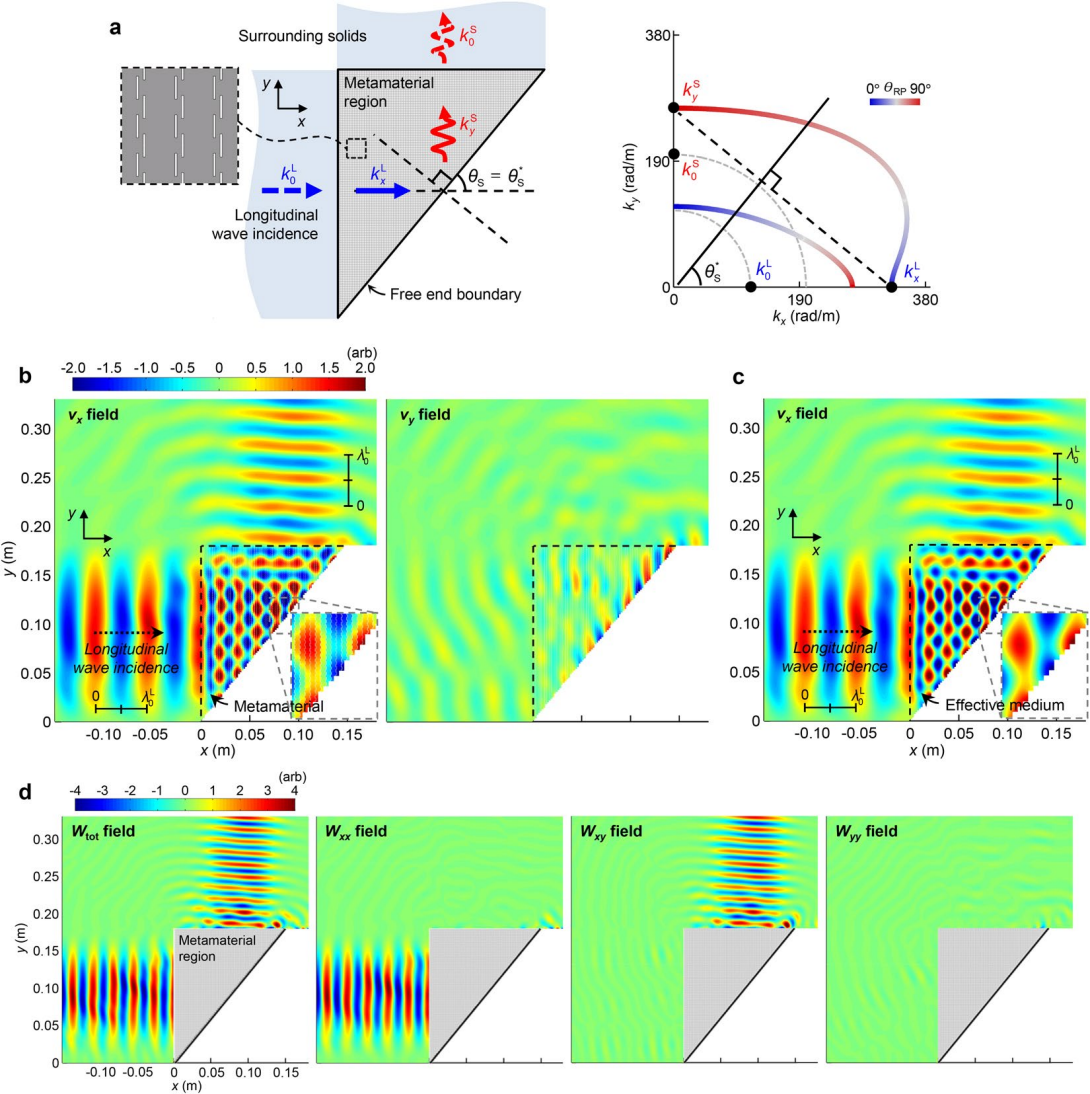
Application of the proposed metamaterial to a wave mode converting wedge. (**a**) Sketch of the designed wave mode converter geometry (left) and the EFCs of the constituent metamaterial with line colors that represent ${\theta }_{{\rm{RP}}}$ (right). The converter is built in a right-angled wedge form (shown in gray) by filling the entire area with a periodic array of the metamaterial. The inclined boundary of the converter at ${\theta }_{{\rm{S}}}$ is free-end while the other boundaries directly meet surrounding solids like aluminum. (EFCs of aluminum are indicated with gray dashed lines). A plane-like longitudinal wave is assumed to be incident to the left boundary of the converter along the *x*-direction from surrounding aluminum. The wavevectors involved in the simulations are predicted in the EFC plot. Time-harmonic simulations are conducted at $f=100\,{\rm{kHz}}$ by using (**b**) the detailed metamaterial model (left: *x*-, right: *y*-directional velocity fields) and (**c**) the effective medium model (*x*-directional velocity field). (**d**) The same simulation results from the detailed model are also represented by the strain energy density fields: total (${W}_{{\rm{tot}}}={W}_{xx}+{W}_{xy}+{W}_{yy}$) and partial (${W}_{xx}={\sigma }_{xx}{\varepsilon }_{xx}$, ${W}_{xy}=2{\sigma }_{xy}{\varepsilon }_{xy}$, ${W}_{yy}={\sigma }_{yy}{\varepsilon }_{yy}$). Only the fields outside the converter are presented.

In Fig. 6a, the schematic configuration of our proposed converter is illustrated. The converter is built in a right-angled wedge form (shown in gray) by filling the entire area with a periodic array of the metamaterial of the slit length $h=1.08\,{\rm{mm}}$. For a fundamental study, we assume that the converter is surrounded by semi-infinite homogenous aluminum except for the incline set to be free-end. A plane-like longitudinal wave is incident to the left boundary of the converter along the *x*-direction from the surrounding aluminum. In the actual design, the incline cannot be linear due to the rectangular shape of the unit cell, but it can be considered approximately linear in the long-wavelength limit.

The EFCs of the constituent metamaterial (shown on the right in Fig. 6a) may be helpful to understand the mechanism of the proposed converter. The EFCs of the metamaterial are indicated in color to represent *θ*_RP_ while those of surrounding aluminum are represented by gray dashed lines. The wavevector of an incident longitudinal wave, in aluminum, is defined as $({k}_{x},\,{k}_{y})=({k}_{0}^{{\rm{L}}},\,0)$ under the plane wave assumption where ${k}_{0}^{{\rm{L}}}$ is marked in the EFC plot. The incident wave should penetrate the left boundary of the converter while maintaining the longitudinal polarization since the incident wave dominantly exerts compressional stress on the interface while possessing negligible tangential momentum. The wavevector of the transferred wave, therefore, can be expressed as $({k}_{x}^{{\rm{L}}},\,0)$ where ${k}_{x}^{{\rm{L}}}$ unusually lies on the outer EFC due to the polarization anomaly of the metamaterial. At the free-end incline of the converter, the wavevector should be drastically changed by the wave reflection. Thus, the slope angle is specifically set to be ${\theta }_{{\rm{S}}}={\theta }_{{\rm{S}}}^{\ast }={90}^{\circ }-{\rm{atan}}\sqrt{{C}_{66}^{{\rm{eff}}}/{C}_{11}^{{\rm{eff}}}}$ where ${C}_{11}^{{\rm{eff}}}$ and ${C}_{66}^{{\rm{eff}}}$ are the effective normal and shear stiffnesses, respectively, of the metamaterial at $f=100\,{\rm{kHz}}$ with ${C}_{11}^{{\rm{eff}}}=9.12\,{\rm{GPa}}$, ${C}_{66}^{{\rm{eff}}}=13.47\,{\rm{GPa}}$, and ${\theta }_{{\rm{S}}}^{\ast }={50.5}^{\circ }$ (similar ${\theta }_{{\rm{S}}}^{\ast }$ will be obtained even though the effective stiffnesses are selected at other low frequencies). By drawing a straight line perpendicular to the incline from the incident wavevector, $({k}_{x}^{{\rm{L}}},\,0)$ (illustrated by black dashed lines), we can predict the wave reflection since only the wave components meeting the line satisfy the tangential momentum conservation rule at the incline^1,26,27^. In the present case, only the wavevector $(0,\,{k}_{y}^{{\rm{S}}})$ should be involved in the reflection. The corresponding wave is purely shear even though it shares the same outer EFC branch as the longitudinal wave with $({k}_{x}^{{\rm{L}}},\,0)$. The wave mode conversion from longitudinal to shear mode can be surprisingly achieved during the reflection. Ideally, no energy loss occurs during the conversion, because unwanted multiple wave reflections can be avoided. By elaborately tailoring the dispersion property of the metamaterial, the desired EFC profiles for the pure conversion were successfully derived in the design. The converted shear wave should be transferred to the upper interfacing aluminum without additional mode conversion for the same reason as in the previous input case. Consequently, the purely shear wave with $(0,\,{k}_{0}^{{\rm{S}}})$ is obtained at the output region.

In order to validate the designed converter, the time-harmonic simulations are conducted. The converter dimension is set as ${D}_{{\rm{L}}}=99\times L=17.8\,{\rm{cm}}$ (*L* = lattice constant of the metamaterial) for the left edge while ${D}_{{\rm{U}}}=81\times L=14.6\,{\rm{cm}}$ for the upper edge, leading to the ${\theta }_{{\rm{S}}}={\rm{atan}}({D}_{{\rm{L}}}/{D}_{{\rm{U}}})={50.7}^{\circ }$ slope. The slope angle virtually matches the targeted ${\theta }_{{\rm{S}}}^{\ast }={50.5}^{\circ }$. Except for the outside region bordering the incline, the converter is enclosed with a homogeneous aluminum. A longitudinal wave source with the Gaussian distribution in the *y*-direction is excited in the left aluminum region along the *x*-direction. The perfectly matched layers were used at the far-field region (not shown here) to prevent any other reflection not from the converter.

The simulation results at $f=100\,{\rm{kHz}}$ are represented by the *x*- and *y*-directional velocity fields (${v}_{x}$ and ${v}_{y}$) in Fig. 6b. (The results for other frequencies are reported in Supplementary H). The same simulations are also conducted with the effective medium model of the converter and the results for the ${v}_{x}$ field are specifically shown in Fig. 6c. The emitted wave from the converter propagating in the *y*-direction virtually exhibits only the *x*-directional particle motions with negligible *y*-directional motion. The observation indicates that the emitted wave is virtually shear as confirmed by the shorter wavelength of the output wave compared to that of the incident longitudinal wave (${\lambda }_{0}^{{\rm{L}}}=5.43\,{\rm{cm}}$; see the scale bar) where shear wavelength in aluminum is supposed to be ${\lambda }_{0}^{{\rm{S}}}=3.14{\rm{cm}}=0.58\times {\lambda }_{0}^{{\rm{L}}}$. The pure conversion from longitudinal to shear modes was successfully obtained as predicted in Fig. 6a. The same results are obtained using the effective medium model. In Fig. 6d, the results are also represented by the strain energy density fields: total field (${W}_{{\rm{tot}}}={W}_{xx}+{W}_{xy}+{W}_{yy}$) and each partial field (${W}_{xx}={\sigma }_{xx}{\varepsilon }_{xx}$, ${W}_{xy}=2{\sigma }_{xy}{\varepsilon }_{xy}$, and ${W}_{yy}={\sigma }_{yy}{\varepsilon }_{yy}$ where ${\sigma }_{xx}$ (${\varepsilon }_{xx}$) and ${\sigma }_{yy}$ (${\varepsilon }_{yy}$) are the normal stress (strain) for the *x*- and *y*-directions, respectively, as well as ${\sigma }_{xy}$ (${\varepsilon }_{xy}$), the shear stress (strain)). The results show that both the incident and reflected waves in the input region are essentially the purely longitudinal waves while the transmitted wave in the output region are the purely shear wave. Other wave modes do not develop in the surrounding media.

It may be worth remarking on the conversion efficiency of the converter. Because negligible energy loss occurs during the wave reflection (conversion) procedure, the dominant factors that determine the conversion efficiency should be the impedance-matching conditions at the left and upper interfaces of the converter between the constituent metamaterial and surrounding media. Furthermore, the conversion efficiency may be also somewhat dependent on the working frequency under the time-harmonic wave input. At $f=100\,{\rm{kHz}}$, the transmittance of the shear wave in the output region is observed to be about 0.60 while the reflectance of the longitudinal wave in the input region is about 0.33. Approximately 7% of the energy loss relative to an incident power is unexpectedly observed, and it seems to be mainly from leaky waves at the upper-right corner of the converter due to the finite effect of the considered system. Nevertheless, a large portion of incident power is successfully converted into the shear wave. The conversion efficiency can possibly be increased more if the metamaterial substrate is properly re-selected for a target specimen or if the geometric dimensions of the converter are properly adjusted according to the working conditions like frequency.

### Validations by experiments

**Figure 7:**
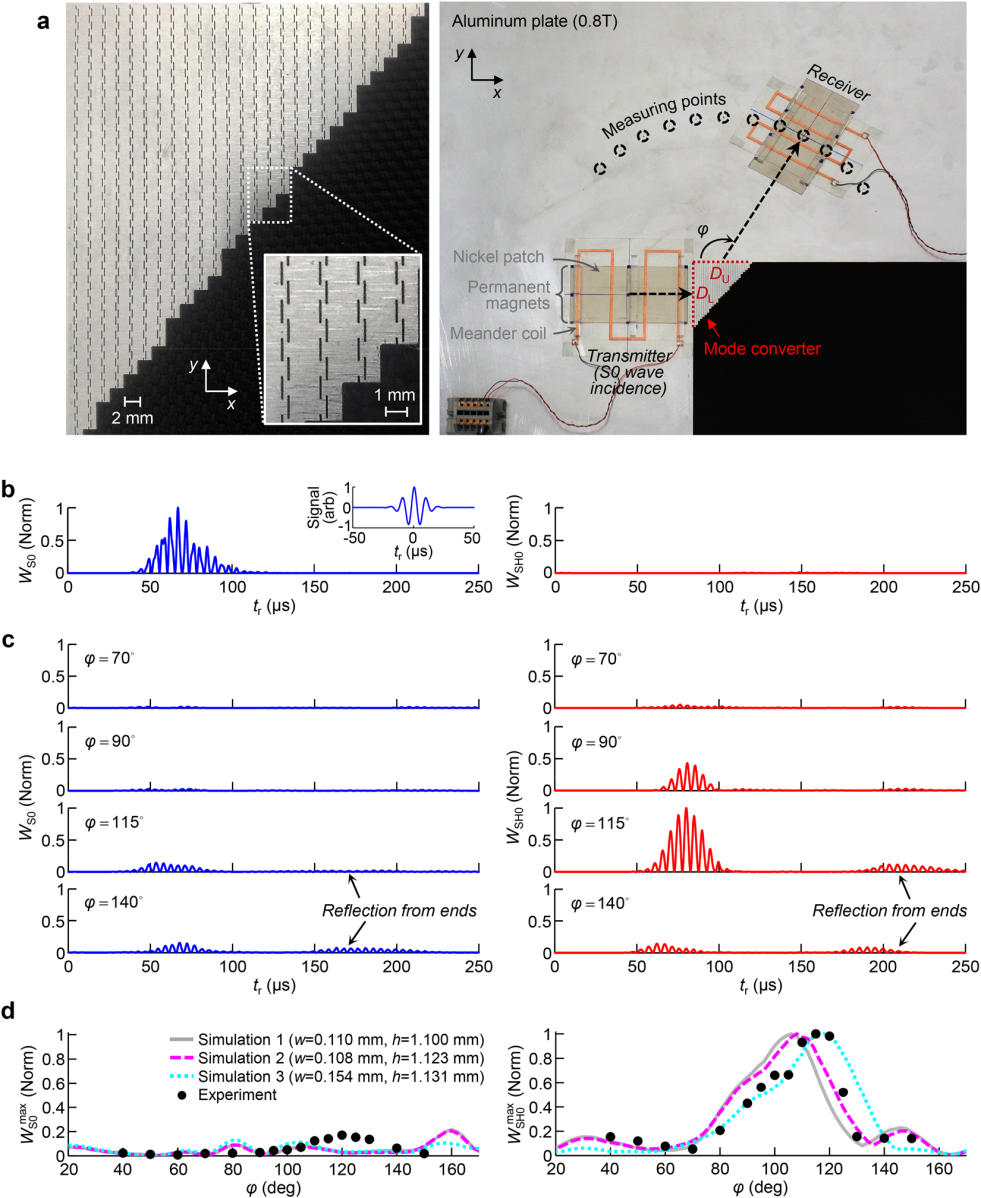
Experimental validations of the fabricated wave mode converting wedge. (**a**) Photo of the fabricated converter in an 8-mm-thick aluminum plate (left) and the experimental setups at $f=100\,{\rm{kHz}}$ (right). Meander-type magnetostrictive transducers consisting of a nickel patch, permanent magnets, and meander coil, are employed to generate and measure the lowest symmetric Lamb wave mode (S0 mode) and the lowest shear-horizontal wave mode (SH0 mode). The dimensions of the fabricated converter are ${D}_{{\rm{L}}}=5.76\,{\rm{cm}}$ for the left edge and ${D}_{{\rm{U}}}=4.68\,{\rm{cm}}$ for the upper edge. (**b**) As reference, the wave generated by the S0-wave transmitter is directly measured without the mode converter in a pure aluminum plate 30 cm away from the transmitter. The measured normal (left) and shear (right) strain energies, ${W}_{{\rm{S0}}}$ and ${W}_{{\rm{SH0}}}$, are shown over time (${t}_{{\rm{r}}}$). The input Gaussian-modulated sinusoidal pulse with the center frequency of $f=100\,{\rm{kHz}}$ is also shown in the inset. (**c**) The measured strain energies, ${W}_{{\rm{S0}}}$ (left) and ${W}_{{\rm{SH0}}}$ (right) of the transmitted wave from the converter, are shown for $\phi ={70}^{\circ }$ (first row), ${90}^{\circ }$ (second), 115° (third), and 140° (fourth) normalized by the maximum value of ${W}_{{\rm{SH0}}}$ at $\phi ={115}^{\circ }$. The second arrival signals in the plots are from the reflected waves at the plate ends, which are not within the scope of this paper. (**d**) The maximum values of ${W}_{{\rm{S0}}}$ (left) and ${W}_{{\rm{SH0}}}$ (right) of the transmitted wave from the converter are measured at each $\phi$ defined as ${W}_{{\rm{S0}}}^{\max }$ and ${W}_{{\rm{SH0}}}^{\max }$, respectively. The black dots represent the experimental results and the time-transient simulation results are also shown. The following different sets of slit width and length for the metamaterial unit cell are considered in the simulations in order to take into consideration fabrication errors with ($w=0.110\,{\rm{mm}}$, $h=1.100\,{\rm{mm}}$) indicated by gray solid lines, (*w* = 0.108 mm, $h=1.123\,{\rm{mm}}$) by magenta dashed lines, and ($w=0.154\,{\rm{mm}}$, $h=1.131\,{\rm{mm}}$) by cyan dotted lines. The experimental and simulation results are normalized by the largest ${W}_{{\rm{SH0}}}^{\max }$ in each case.

To experimentally validate the proposed converter working by polarization anomaly, we fabricated the converter in a 0.8-mm-thick aluminum plate using micro end-milling technology, as shown in Fig. 7a. The tungsten carbide tool used for the fabrication has the diameter of 0.1 mm. It moved horizontally at the speed of 1 mm/s while rotating at 600 rpm. The geometric parameters of the metamaterial unit cell are slightly modified to $L=1.8\,{\rm{mm}}$, $w=0.11\,{\rm{mm}}$, $h=1.10\,{\rm{mm}}$, and $s=0.3\,{\rm{mm}}$ to take into account some fabrication errors in micro-scale machining. (See Supplementary I for more details.) The overall dimension of the converter is greatly reduced to ${D}_{{\rm{L}}}=32\times L=5.76\,{\rm{cm}}$ for the left edge and ${D}_{{\rm{U}}}=26\times L=4.68\,{\rm{cm}}$ for the upper edge compared to that considered in the previous simulations shown in Fig. 6. The dimension is even similar to the longitudinal wavelength scale ${\lambda }_{0}^{{\rm{L}}}=5.43\,{\rm{cm}}$ at $f=100\,{\rm{kHz}}$. Other geometric setups for the slope angle of the free-end incline and the surrounding material of the converter are exactly the same as those in simulations. For the present plate thickness and frequency conditions, the lowest symmetric Lamb wave mode, namely the S0 mode, in a plate behaves as a longitudinal wave in a bulk medium while the lowest shear-horizontal wave mode, namely the SH0 mode, in a plate behaves as a shear wave in a bulk medium^2,28^. Therefore, the analyses and simulations performed for the bulk waves can be validated by the S0 and SH0 wave experiments conducted in a plate. As transducers for the S0 (longitudinal) and SH0 (shear) wave modes, the meander-type magnetostrictive transducers^29,30^ that consist of a nickel patch, permanent magnets, and meander coil are employed. By adjusting the intervals between the adjacent meander coil lines, the transducers can generate and measure only the targeted wave modes. (See Supplementary J for detailed information on the used transducers.) As a longitudinal wave source, the S0 wave transmitter is placed just in front of the left edge of the converter (the distance between the left edge of the converter and the nearest meander line is 1.15 cm). Then, at locations 13 cm away from the upper edge center of the converter, the transmitted waves from the converter are measured by using the S0 and SH0 wave receivers. To measure the transmitted angle ($\phi$) of the waves, the measuring location is also varied for ${40}^{\circ }\le \phi \le {150}^{\circ }$. Note that the used S0 and SH0 wave transducers were calibrated through additional experiments. (In Supplementary K, the detailed calibration procedure is presented.)

For reference, we first performed an experiment without the converter in a pure aluminum plate. The wave generated by the S0 wave transmitter is measured by the S0 and SH0 wave receivers 30 cm away from the transmitter. In Fig. 7b, the normal strain energy ${W}_{{\rm{S0}}}$ (left) measured by the S0 receiver and the shear strain energy ${W}_{{\rm{SH0}}}$ (right) measured by the SH0 receiver are shown with respect to the time (${t}_{{\rm{r}}}$) normalized by the maximum value of ${W}_{{\rm{S0}}}$. As an input signal, the modulated Gaussian pulse with the center frequency of $f=100\,{\rm{kHz}}$ is used (see inset). The generated wave is observed to exhibit dominant ${W}_{{\rm{S0}}}$ with negligible ${W}_{{\rm{SH0}}}$, confirming the excellent sensitivities of the used transducers for the targeted wave modes. The experiments were conducted with the fabricated mode converter. In Fig. 7c, the normal (${W}_{{\rm{S0}}}$) and shear (${W}_{{\rm{SH0}}}$) strain energies, particularly measured at $\phi ={70}^{\circ }$ (first row), 90° (second), 115° (third), and 140° (fourth) are presented (normalized by the maximum value of ${W}_{{\rm{SH0}}}$ at $\phi ={115}^{\circ }$). The shear strain energy dominantly appears in the transmitted waves relative to the normal strain energy. When further examining the spatial distributions of the normal and shear strain energies along $\phi$ (see black dots in Fig. 7d), it can be confirmed that the transmitted waves from the converter are dominantly shear at every $\phi$. ${W}_{{\rm{S0}}}^{\max }$ and ${W}_{{\rm{SH0}}}^{\max }$ indicate the maximum values of normal and shear strain energies of the transmitted wave of interest normalized by ${W}_{{\rm{SH0}}}^{\max }$ at $\phi ={115}^{\circ }$. In the same plots, the experimental results were compared to the simulation results from the time-transient analysis. Three different metamaterial geometries are considered in the simulations to take the fabrication errors into account: the original designed geometry with $w=0.110\,{\rm{mm}}$, $h=1.100\,{\rm{mm}}$ (indicated by gray solid lines) as well as actual fabricated geometries with the small and large fabrication errors as $w=0.108\,{\rm{mm}}$, $h=1.123\,{\rm{mm}}$ (magenta dashed lines) and $w=0.154\,{\rm{mm}}$, $h=1.131\,{\rm{mm}}$ (cyan dotted lines), respectively. (Other geometric parameters had relatively negligible fabrication error so their variations are not considered here. See Supplementary L for more details.) The distributions of ${W}_{{\rm{S0}}}^{\max }$ and ${W}_{{\rm{SH0}}}^{\max }$ from the simulations are also normalized by the maximum value of ${W}_{{\rm{SH0}}}^{\max }$ in each case. The radiation patterns of the transmitted wave estimated from the simulations agree favorably with those from experiments when considering actual fabrication errors.

The main reason why the transmitted waves from the converter show the peak shear strain energy at around $\phi ={115}^{\circ }$ and not at $\phi ={90}^{\circ }$ even in the simulations are due to the finite effects in the converter. Because the dimensions of the fabricated converter are quite small even comparable to a wavelength scale, the finite effects should be quite significant. The wavevectors inside the converter cannot be ideally described as in Fig. 6a and the involving wave phenomena are slightly deviated from predictions. Nevertheless, as well validated in the simulations and experiments, the core physics of the designed converter are found to be valid.

## Discussions

In this paper, we realized the polarization anomaly phenomenon for elastic waves by special off-centered double-slit metamaterials for the first time. The key feature of the metamaterial is in its extraordinary flexibility for dilatation in the perpendicular direction to the slits even compared to that for shear deformation as opposed to the ordinary stiffness for the dilation in the orthogonal direction. The novelty originates from the special off-centered slit arrangement, not relying on any local resonance. The metamaterial retains the peculiar material anisotropy fairly well throughout the low frequency regime even from the static limit. As a novel application of the proposed metamaterial, we propose an elastic wave mode converting wedge that can purely transform an incident longitudinal mode into a shear mode. Due to the high programmability of the metamaterial in the dispersion property, the desired equi-frequency contour curvature for the pure conversion was elaborately tailored in the design. The efficient and robust conversion physics of the converter was successfully validated by numerical simulations and experiments.

When considering the polarization anomaly, unprecedented wave behaviors are brought up in terms of the dispersion. The frequency-dependent dispersion, spatial dispersion, and polarization properties are used so that the proposed metamaterial makes a significant impact in manipulating elastic waves. The metamaterial will open up new opportunities to utilize the polarization anomalous phenomenon for novel applications.

## Methods

### Fabrication

The metamaterial-based wave mode converter was fabricated through vertical milling. End mills that cover the micro-scale machining are used as a cutting tool.

### Experimental setups

The meander-type magnetostrictive transducers, which are used in the experiments, consist of a magnetostrictive nickel patch, a meander coil, and multiple permanent magnets. The dimensions of a nickel patch are $9.4\times 5\,{{\rm{cm}}}^{2}$ with 0.15 mm thickness. Right over the patch, a 4-line meander coil (3 mm for each line width) and multiple neodymium magnets ($3\times 3\times 25\,{{\rm{mm}}}^{3}$) were installed (see Fig. 7a for the overall figure). The nickel patch was tightly bonded onto a plate by double-sided adhesive tape. More details on the meander-type magnetostrictive transducers can be seen in Supplementary J.

A function generator (33250 A, Agilent, Santa Clara, CA) was used to transmit a modulated Gaussian pulse to a transmitting transducer through a power amplifier (AG1017L, T&C Power Conversion, Rochester, NY) for wave excitation. The measured signals by a receiving transducer were transmitted to a pre-amplifier (SR560, Stanford Research Systems, Sunnyvale, CA) and then recorded by an oscilloscope (WaveRunner 104MXi-A, LeCroy, Chestnut Ridge, NY).

### Simulations

All the numerical simulations in this paper were performed by using the structural mechanics module in COMSOL Multiphysics 3.5a.

### Data availability

The data in this study are available on request from the authors.

## Electronic supplementary material

Supplementary information
