# Supplementary material for: Off-centered Double-slit Metamaterial for Elastic Wave Polarization Anomaly
Source: Sci Rep. 2017 Nov 13;7:15378. doi: 10.1038/s41598-017-15746-2 (PMC5684245; doi:10.1038/s41598-017-15746-2)
Supplement: Supplementary file 1 — Supplementary information [file 41598_2017_15746_MOESM1_ESM.pdf]

Supplement information for

Off-centered Double-slit Metamaterial for Elastic Wave Polarization Anomaly

Hyung Jin Lee[1], Je-Ryung Lee[2], Seung Hwan Moon[2], Tae-Jin Je[2],

Eun-chae Jeon[2], Kiyean Kim[3], and Yoon Young Kim[4,*]

[1]Institute of Advanced Machines and Design, Seoul National University, 599 Gwanak-ro, Gwanak-gu, Seoul 151-742, South Korea, [2]Korea Institute of Machinery and Materials (KIMM), 156 Gajeongbuk-ro, Yuseong-gu, Daejeon, South Korea, [3]School of Mechanical and Aerospace Engineering, Seoul National University, 599 Gwanak-ro, Gwanak-gu, Seoul 151-742, South Korea, [4]WCU Multiscale Mechanical Design Division, School of Mechanical and Aerospace Engineering, Seoul National University, 599 Gwanak-ro, Gwanak-gu, Seoul 151-742, South Korea. Correspondence and request for materials should be addressed to Y.Y.K. (e-mail: yykim@snu.ac.kr)

Supplementary A. Polarization characteristics for high anisotropy factor

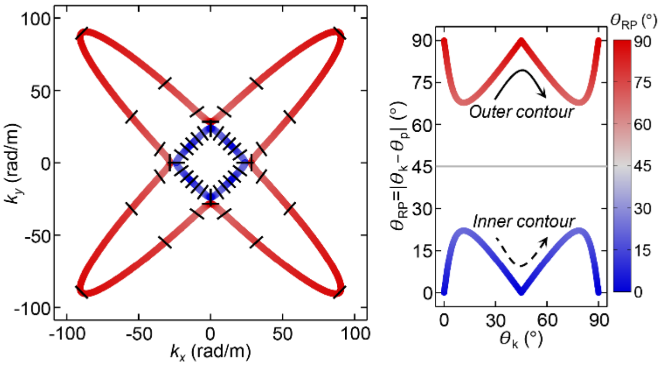

Figure S1. Equi-frequency contours (EFCs) and relative polarization orientation, $\theta_{RP} = |\theta_P - \theta_k|$ (θ_P : polarization orientation, θ_k: wavevector orientation), at 30 kHz for an arbitrary solid with a high anisotropy factor. The material properties used for the plot are $\rho = 4260\ \text{kg/m}^3$, $C_{11} = C_{22} = 26.60 \times 10^{10}\ \text{Pa}$, $C_{12} = 24.71 \times 10^{10}\ \text{Pa}$, and $C_{66} = 18.86 \times 10^{10}\ \text{Pa}$.

Here, we would like to show that the ordinary polarization characteristics in solids, (quasi-)longitudinal mode for the inner EFC branch and (quasi-)shear mode for the outer branch, are invariant even for a high anisotropy factor. For instance, the anisotropy factor of Rutile (see Fig. 1b for the original property) is increased by 400 % and the relative polarization orientation, $\theta_{RP} = |\theta_P - \theta_k|$ are investigated in Fig. S1. The anisotropy factor used here is $A = 2C_{66}/(C_{11} - C_{22}) = 20$ where $C_{11} = C_{22} = 26.60 \times 10^{10}\ \text{Pa}$, $C_{12} = 24.71 \times 10^{10}\ \text{Pa}$, and $C_{66} = 18.86 \times 10^{10}\ \text{Pa}$ ($\rho = 4260\ \text{kg/m}^3$).

As seen in the EFCs, a significant anisotropy is found. The polarization characteristics, however, are virtually the same as the original ones still following the usual characteristics in (quasi-)longitudinal for the inner EFC and (quasi-)shear for the outer EFC. The anisotropy factor itself does not correlate with the polarization anomaly.

Supplementary B. Material property of $C_{11} < C_{66} < C_{22}$ for the polarization anomaly.

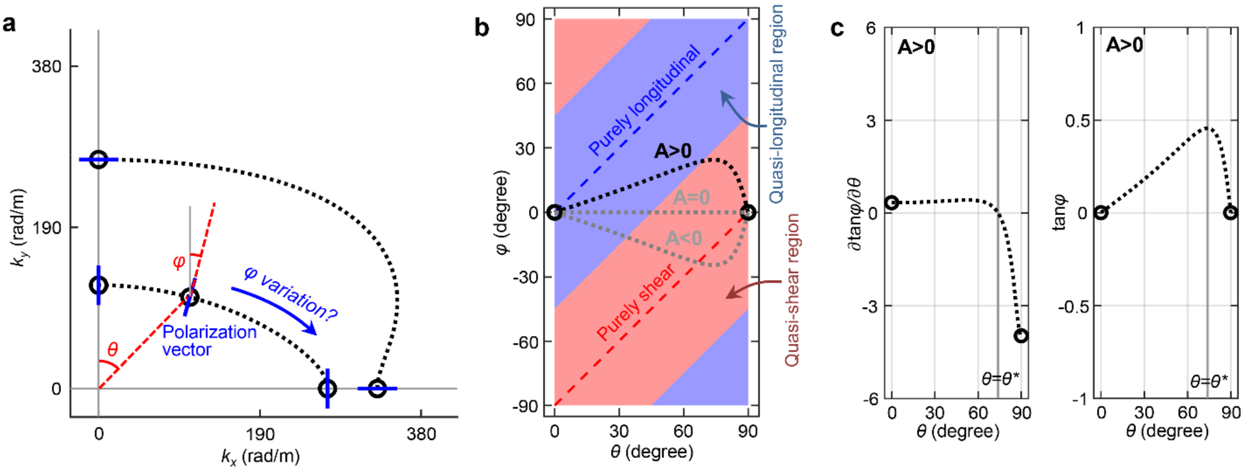

Figure S2. (a) Equi-frequency contours (EFCs) of the metamaterial with the slit length of $h = 1.08$ mm at $f = 100$ kHz, shown in Fig. 3c in the manuscript, are indicated with the black dotted lines while the polarization angles at certain wavevector angles including $\theta = 0$ and $\pi/2$, with the blue solid poles. (b) Variations of polarization angle φ, which belongs to the inner EFC branch, as well as (c) the variations of $\partial \tan\varphi/\partial\theta$ and $\tan\varphi$ are investigated as the wavevector angle θ changes from 0 to $\pi/2$. In **b**, the φ variation for $A > 0$ is indicated with the black dotted line while those for $A = 0$ and $A < 0$, with the gray dotted lines. Quasi-longitudinal and quasi-shear regions are illustrated in blue and red, respectively, and purely longitudinal and purely shear regions, by blue and red dashed lines, respectively.

The material property condition of $C_{11} < C_{66} < C_{22}$ always leads to the polarization anomaly. The reason can be found by analytically investigating the change of polarization along an EFC branch under the material property condition as the wavevector changes, as done by K. Helbig *et al.* [1].

Here, let us skip the detailed derivation procedure and just adopt the results from [1]. By following their convention in defining the material properties such that

$$c_{11} \equiv C_{11}/C_{66}\,,\;\; c_{22} \equiv C_{22}/C_{66}\,,\;\; c_{12} \equiv C_{12}/C_{66}\,,\;\; A \equiv c_{12}+1\,,\;\; B \equiv c_{11}-1\,,\;\; C \equiv c_{22}-1\,,$$

one can express the polarization angle φ corresponding to the inner (fast) EFC branch as:

$$\tan\varphi = \frac{A\sin\theta}{2/S_1^2 - 2 - B - B\cos\theta} \tag{S1}$$

where θ denotes the wavevector angle and the coefficient S_1 is defined as

$$2/S_1^2 \equiv X + 1 + D$$

with

$$X \equiv 1 + (C+B)/2 + \{(C-B)/2\}\cos 2\theta > 0\,,$$

and

$$D = \sqrt{\left[(C-B)/2 + \{(C+B)/2\}\cos 2\theta\right]^2 + \left(A\sin 2\theta\right)^2}\,.$$

Note that both the polarization angle φ and the wavevector angle θ are measured from the y-axis (see Fig. S2a). Because we are interested in investigating how polarization changes along a EFC branch, the variation of polarization angle φ that corresponds to the inner branch with respect to the variation of θ can be examined here. (Note that we will only consider it because the polarization angle for the outer branch is simply $\varphi + \pi/2$.) Specifically, we examine $\partial(\tan\varphi)/\partial\theta$:

$$\frac{\partial \tan\varphi}{\partial\theta} = \frac{A\left[(C+B)+(C-B)\cos 2\theta\right]}{D\left[(C-B)/2 + \{(C+B)/2\}\cos 2\theta + D\right]}\,. \tag{S2}$$

Because D is not negative and $D > \left|(C-B)/2 + \{(C+B)/2\}\cos 2\theta\right|$ for $0 < \theta < \pi/2$, the denominator of Eq. (S2) is always positive for $0 < \theta < \pi/2$.

Let us now investigate the φ variation under the material condition of $C_{11} < C_{66} < C_{22}$ (equivalently, $B < 0$, $C > 0$) as θ changes from 0 to $\pi/2$. From Eq. (S1), we can find $\varphi = 0$ when θ approaches 0 and $\pi/2$, as illustrated in the EFC space in Fig. S2a where the blue solid poles represent the polarization vectors. The polarization angles are also indicated in the θ-φ space with the black circles in Fig. S2b where the regions of φ corresponding to the quasi-longitudinal and quasi-shear modes are indicated in blue and red, respectively for varying θ's. Those corresponding to the purely longitudinal and purely shear modes are denoted by the blue and red dashed lines, respectively. Because the polarization angles of $\varphi = 0$ at $\theta = 0$ and $\theta = \pi/2$ belong to purely longitudinal and purely shear regions, respectively, φ is supposed to cross from the quasi-longitudinal to quasi-shear region. In other words, the longitudinal-to-shear polarization transition occurs as θ increases from 0 to $\pi/2$. From Eq. (S2), we can see that $\partial\tan\varphi/\partial\theta$ varies continuously as a function of θ for $0 < \theta < \pi/2$ because both the numerator and denominator in Eq. (S2) are continuous while the denominator is always positive. Therefore, both $\tan\varphi$ and φ are continuous in the range of

$0 < \theta < \pi/2$. The polarization anomaly always takes place when $C_{11} < C_{66} < C_{22}$.

Specifically for our metamaterial model with the slit length of $h = 1.08\ \mathrm{mm}$, shown in Fig. 3c in the manuscript, the detailed variations of $\partial \tan\varphi/\partial\theta$ and $\tan\varphi$ are examined in Fig. S2c for $0 < \theta < \pi/2$ while the variation of φ is shown in Fig. S2b (see the black dotted lines in the plots). As previously mentioned, all the parameters continuously vary in the range of $0 < \theta < \pi/2$. The sign of $\partial \tan\varphi/\partial\theta$ is determined by the sign of A as $\mathrm{sign}(\partial \tan\varphi/\partial\theta) = \mathrm{sign}(A)$ for $\theta < \theta^*$ while $\mathrm{sign}(\partial \tan\varphi/\partial\theta) = \mathrm{sign}(-A)$ for $\theta > \theta^*$ where $\theta^* = \mathrm{atan}\sqrt{-C/B}$, as can be derived from Eq. (S2). Therefore, in our case with $A > 0$, $\tan\varphi$ (or φ) gradually increases for $\theta < \theta^*$ and decreases for $\theta > \theta^*$. Although the increasing-decreasing pattern of φ is varied depending on the sign of A $(= C_{12}/C_{66} + 1)$, φ always continuously vary for non-zero A (φ is constant for $A = 0$; see Fig. S2b). This implies that the polarization anomaly occurs regardless of C_{12} if $C_{11} < C_{66} < C_{22}$.

Supplementary C. Programmability of metamaterial in the anomalous polarization regime

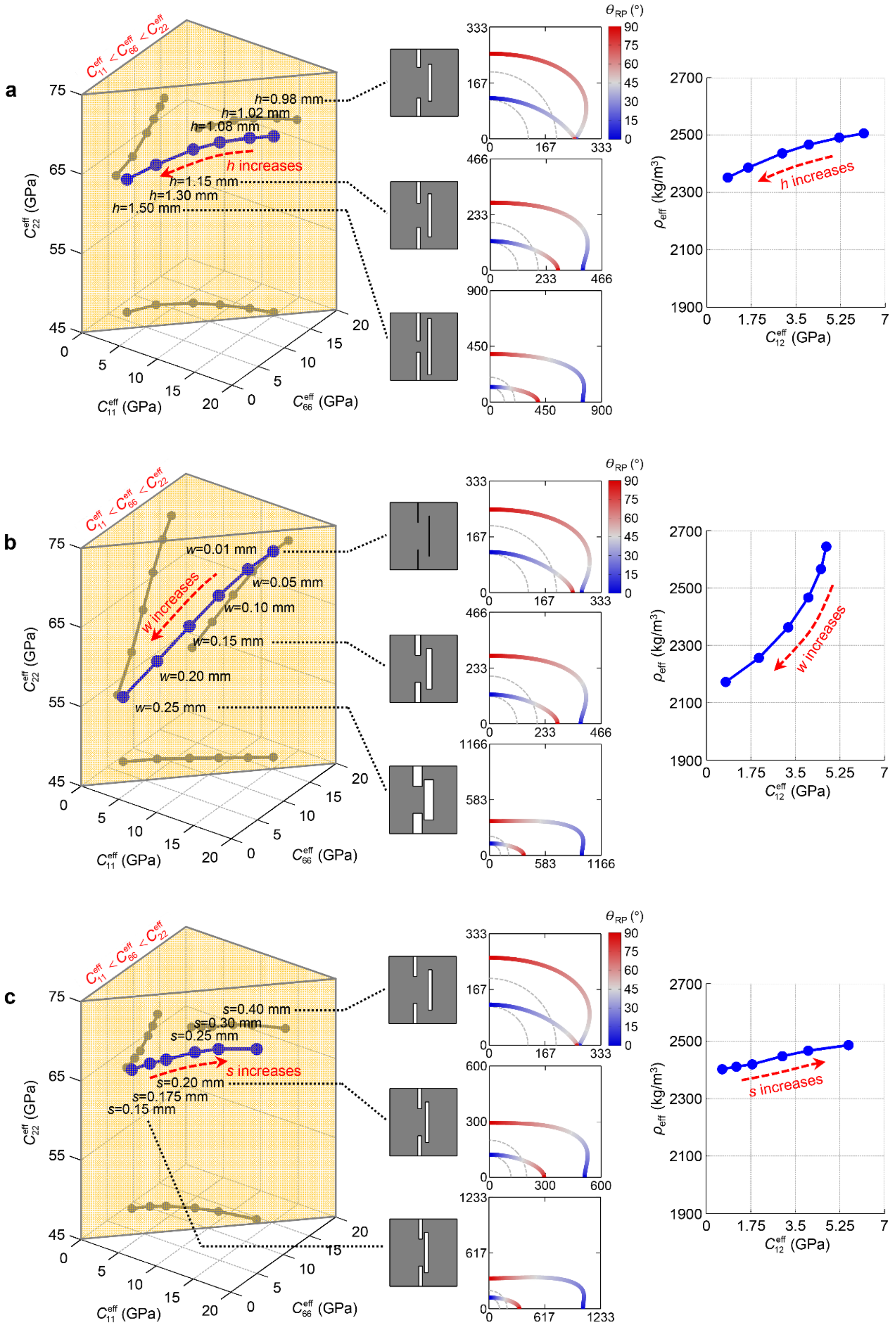

Figure S3. Effective material parameters of the proposed metamaterial at $f = 100$ kHz for a varying geometric parameter: (a) slit length h, (b) slit width w, and (c) separation distance between the adjacent slits s. The other parameters are fixed. The variations in the effective stiffness components C_{11}^{eff}, C_{22}^{eff}, and C_{66}^{eff} are shown on the left while the variations in the other effective material parameters, ρ_{eff} and C_{12}^{eff}, on the right. The variations of the metamaterial configuration and the corresponding EFCs are also presented (middle).

Here, we would like to show how the wide range of effective properties can be tailored by varying the geometric parameters of the proposed metamaterial. In Fig. S3a-c, as a varying parameter, the slit length *h*, the slit width *w*, and the separation distance between the adjacent slits *s* are considered, respectively. The other parameters are fixed. The effective material parameters as well as the EFCs of the metamaterials are calculated at 100 kHz for these varying parameters. In the plot for C_{11}^{eff}, C_{22}^{eff}, and C_{66}^{eff} (left), the anomalous polarization regime that satisfies $C_{11}^{\text{eff}} < C_{66}^{\text{eff}} < C_{22}^{\text{eff}}$ is indicated in yellow.

From the results, one can find that the proposed metamaterial achieves quite a wide range of effective material parameters by varying the geometric parameters. In particular for the effective stiffness C_{11}^{eff}, the metamaterial exhibits an excellent tailoring capability even to a very small value. Different varying sensitivities of the effective material parameters were found in each case. These observations clearly confirm the excellent programmability of the metamaterial on the polarization anomaly.

Supplementary D. Achievement of extremely anisotropic stiffness by the off-centered double-slit arrangement

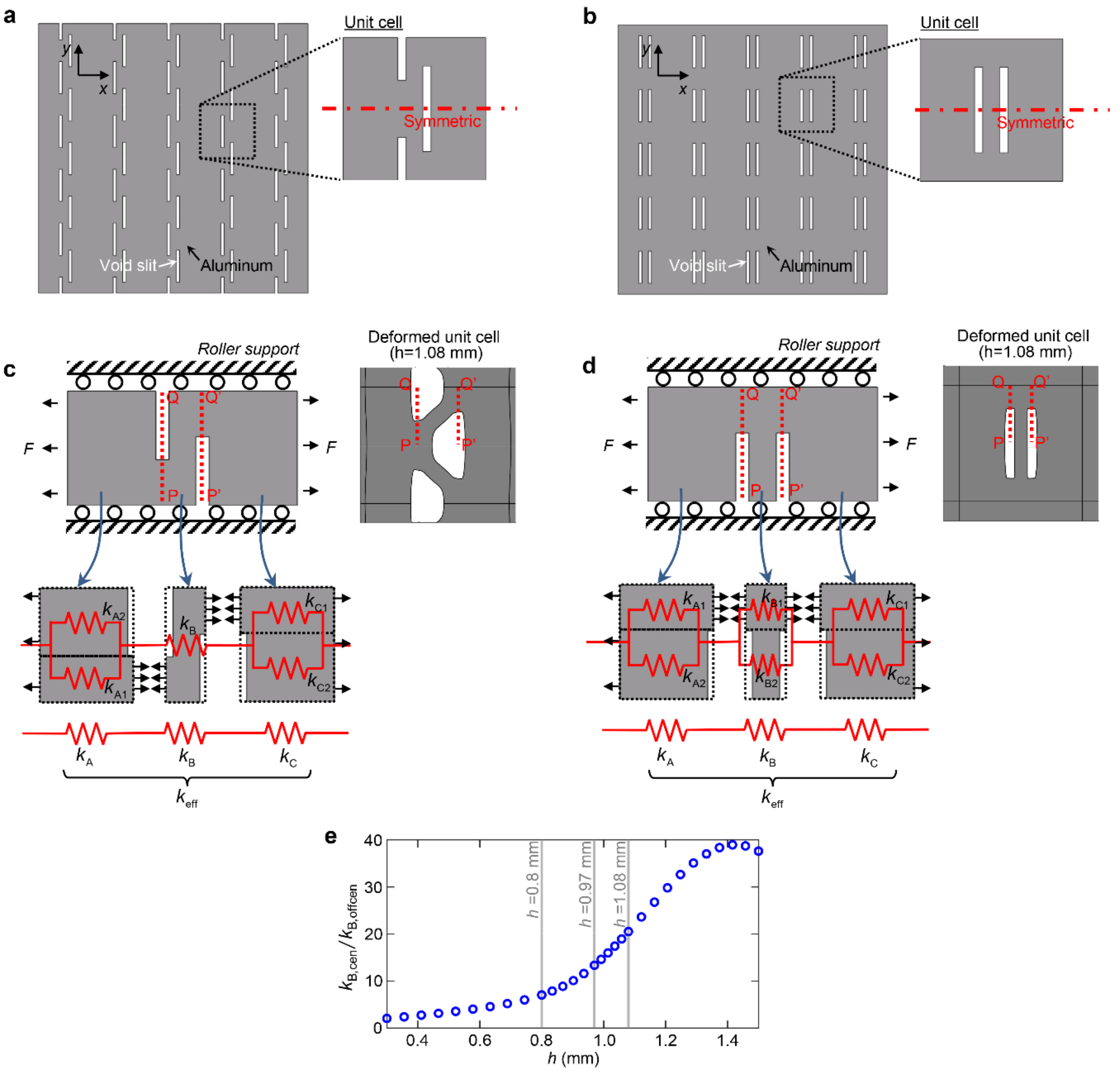

Figure S4. Schematic configurations of (a) the proposed off-centered double-slit metamaterial and (b) the conventional double-slit metamaterial. (c) For the off-centered case, the effective dilatational stiffness in the x-direction is described by the lumped stiffness model for the half of the unit cell. The local stiffnesses are represented by the equivalent dilatational spring constants k_A, k_B, and k_C for the left, center, and right unit cell regions, respectively, where k_A (k_C) is the parallel combination of k_{A1} and k_{A2} (k_{C1} and k_{C2}) for the non-slit and slit regions, respectively. (d) The lumped stiffness model is described for the centered (conventional) case by using k_N (N = A, B, C) where each constant k_N is the parallel combination of k_{N1} and k_{N2}, respectively. The lumped models are derived considering the actual unit cell deformations (see the right in **c** and **d**) (e) The ratio of k_B for the centered and

off-centered cases is estimated by numerically calculating the actual deformations of the corresponding structural components for the varying slit lengths (h).

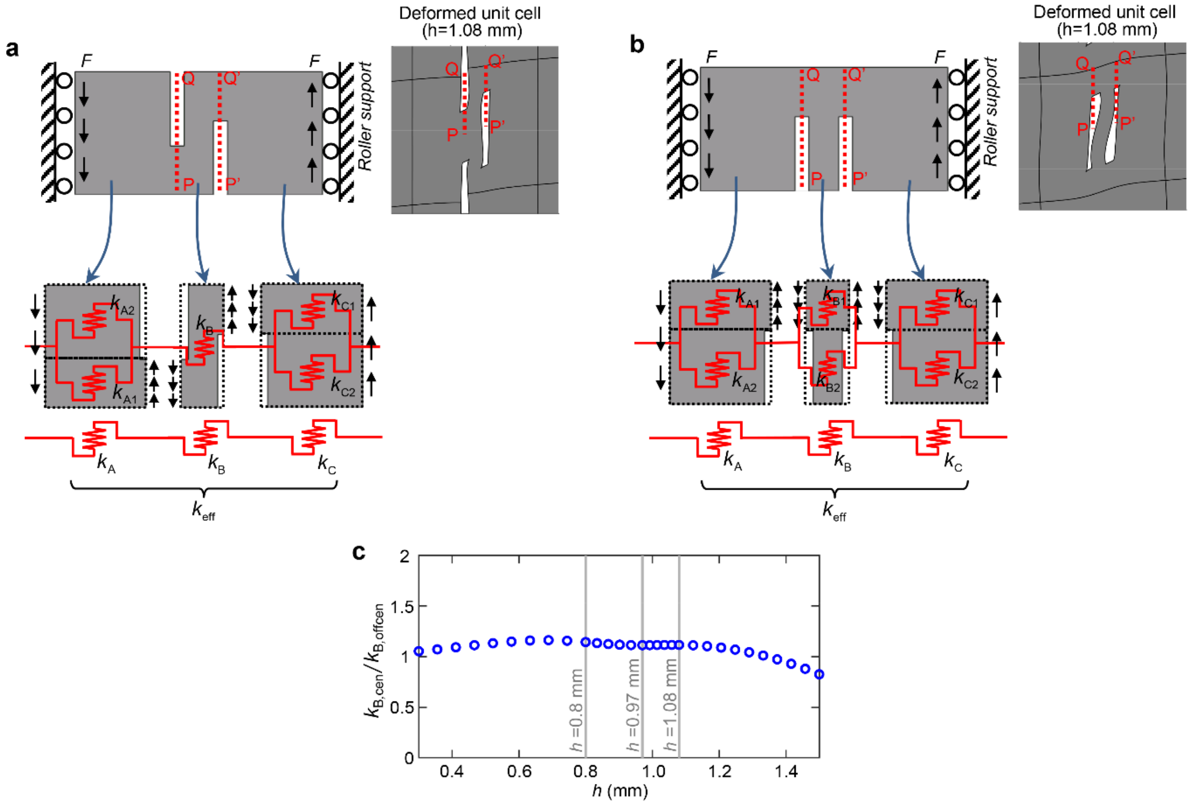

Figure S5. (a) For the off-centered metamaterial case, the effective shear stiffness is described by the lumped stiffness model for the half of the unit cell. The local stiffnesses are represented by the equivalent shear spring constants k_{A} , k_{B}, and k_{C} for the left, center, and right unit cell regions, respectively, where k_{A} (k_{C}) is the parallel combination of k_{A1} and k_{A2} (k_{C1} and k_{C2}) for the non-slit and slit regions, respectively. (b) The lumped stiffness model is described for the centered (conventional) case by using k_N ($N = \mathrm{A, B, C}$) where each constant k_N is the parallel combination of k_{N1} and k_{N2}, respectively. The lumped models are derived considering the actual unit cell deformations (see the right in **a** and **b**) (c) The ratio of k_{B} for the centered and off-centered cases is estimated by numerically calculating the actual deformations of the corresponding structural components for the varying slit lengths (h).

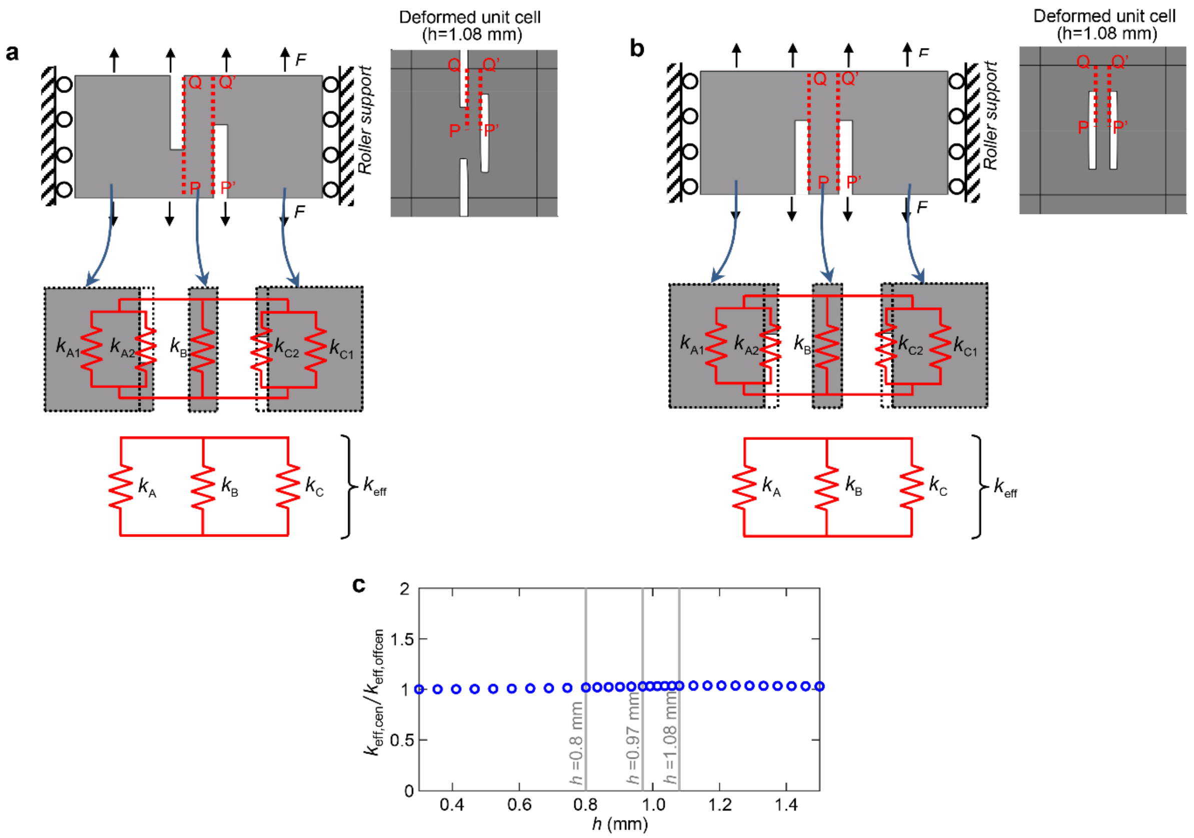

Figure S6. For the (a) off-centered and (b) centered (conventional) metamaterial cases, the effective dilatational stiffnesses in the y-direction are described by the lumped stiffness models for the halves of the unit cells. The local stiffnesses are represented by the equivalent dilatational spring constants k_A, k_B, and k_C for the left, center, and right unit cell regions, respectively, where k_A (k_C) is the parallel combination of k_{A1} and k_{A2} (k_{C1} and k_{C2}) for the non-slit and slit regions, respectively. The lumped models are derived considering the actual unit cell deformations (see the right in **a** and **b**) (c) The ratio of overall effective spring constants (k_{eff}) for the two models are numerically calculated for the varying slit lengths (h).

To explain better why the proposed "off-centered" double slit metamaterial is capable to achieve an extremely low C_{11} with relatively high C_{66} and C_{22} while the conventional double-slit metamaterial is not, we may adopt a simplified lumped element model as explained below.

To model the effective stiffness of the off-centered metamaterial unit cell, we consider two possible combinations of lumped local stiffnesses, one in series and the other in parallel. The lumped stiffness model is schematically described in Figs. S4, S5 and S6. We will begin with Fig. S4 for the dilatational (or, longitudinal) deformation in the x-direction, which governs the behavior of C_{11}. The model for the off-centered configuration (Fig. S4a) is shown in Fig. S4c

for the upper half of the unit cell, and it is compared with the model for the conventional configuration (Fig. S4b) in Fig. S4d. For the derivation, the actual deformed shapes of the unit cells for the x-directional dilatational load (see the right in Figs. S4c and S4d) are considered. In the figures, the local stiffness belonging to the left region of the P-Q line is represented by the stiffness k_{A} of an equivalent spring while the stiffness of the center region between the P-Q and P'-Q' lines and that of the right region of the P'-Q' line can be presented by k_{B} and k_{C} of equivalent springs, respectively. As a first-order approximation, one may model $k_N = k_{N_1} + k_{N_2}$ (N = A, B, C) as illustrated in the figures. From the illustrated model, one can see that the central part of the off-centered case experiences bending deformation, which makes its effective stiffness much smaller than the stiffness for axial deformation. In this case, the corresponding equivalent stiffness may be better expressed by k_{B} alone.

When assuming the dilatational stiffnesses for the local regions adjacent to the slits are negligibly small (because free ends do not support an applied load) compared with those for the non-slit regions, k_{A} and k_{C} should be approximately equal to k_{A1} and k_{C1} , respectively, not only in the off-centered case but also in the centered (conventional) case, i.e., $k_{N,\mathrm{offcen}} \approx k_{N1,\mathrm{offcen}} \approx k_{N,\mathrm{cen}} \approx k_{N1,\mathrm{cen}}$ where $N = \mathrm{A, C}$ (the subscripts 'offcen' and 'cen' imply 'off-centered' and 'centered' cases, respectively); a large value of spring constant dominates the combined spring constants in parallel because the effective spring constant in the parallel case is $k_N = k_{N1} + k_{N2}$. Therefore, the key factor that causes the difference in the overall effective spring constants (k_{eff}) between the off-centered and the centered cases is k_{B} , which corresponds to the local stiffness at the center region of unit cell. The constant k_{B} in the off-centered case may be significantly smaller than k_{B} in the centered case (i.e., $k_{\mathrm{B,offcen}} \ll k_{\mathrm{B,cen}} \approx k_{\mathrm{B1}}$) because $k_{\mathrm{B,offcen}}$ represents the bending-dominant stiffness (see the deformed shape in Fig. S4c) while $k_{\mathrm{B,cen}}$ represents the dilatational (compressional) stiffness where the bending stiffness is much weaker than the dilatational stiffness for such a bar-shaped structure. As the slit length h increases, the deforming behavior of the structural component surrounded by the off-centered slits may resemble the bending motion. In Fig. S4e, the ratio of $k_{\mathrm{B,cen}}$ and $k_{\mathrm{B,offcen}}$ is estimated by numerically calculating the actual deformations of the corresponding structural components. We can observe that $k_{\mathrm{B,cen}} / k_{\mathrm{B,offcen}}$ dramatically increases as h increases. In fact, $k_{\mathrm{B,cen}}$ is even 40 times stiffer than $k_{\mathrm{B,offcen}}$ for around $h = 1.4$ mm . This finding shows that the extraordinary dilatational flexibility of the proposed off-centered double-slit metamaterial with large h originates from the significant reduction in the local stiffness at the region surrounded by the adjacent slits due to the bending-dominant deformation in the region. The weak local stiffness dominates the overall effective stiffness since the effective stiffness can be expressed as follows in terms of the equivalent spring

constants in the series combination: $k_{\text{eff}} = 1 \Big/ \left(\dfrac{1}{k_{\text{A}}} + \dfrac{1}{k_{\text{B}}} + \dfrac{1}{k_{\text{C}}} \right) \approx k_{\text{B}}$ where $k_{\text{A}} \approx k_{\text{C}} \gg k_{\text{B}}$.

We also described the lumped stiffness models for the shear deformation in Fig. S5: the model for the off-centered case in Fig. S5a and the model for the centered (conventional) case in Fig. S5b. The actual deformed shapes of unit cells are considered to model the lumped local stiffnesses. The upper halves of the unit cells are divided into three parts as in the previous analyses. The corresponding local stiffnesses are represented by equivalent shear springs, k_{A} , k_{B}, and k_{C}, respectively. Each spring constant also can be considered as the parallel sum of k_{N1} and k_{N2} ($N = \text{A, B, C}$), which correspond to the non-slit and slit regions, respectively. The local stiffness for the center region in the off-centered case, however, is described with k_{B} alone because a dilation motion in the y-direction is dominant throughout the region in the case.

As previously mentioned, the local stiffnesses in the regions adjacent to the slits may be negligible compared with those in the non-slit regions (because free ends do not support an applied load) so that we can also assume $k_{N,\text{offcen}} \approx k_{N1,\text{offcen}} \approx k_{N,\text{cen}} \approx k_{N1,\text{cen}}$ for $N = \text{A, C}$. Therefore, the constant k_{B} is a sole factor that possibly leads to the difference in the overall effective spring constants (k_{eff}) between the off-centered and the centered cases, just as in the previous dilatational case. In the present off-centered case, on the other hand, the dominant motion that develops in the center region for large h is a y-directional dilation (see the deformed shape in Fig. S5a). Therefore, its corresponding stiffness will be larger than the bending-dominant stiffness observed in the previous case. In Fig. S5c, the detailed ratio of $k_{\text{B,cen}}$ and $k_{\text{B,offcen}}$ is numerically calculated. Throughout the h range of interest, $k_{\text{B,offcen}}$ is observed fairly comparable to $k_{\text{B,cen}}$. This finding supports the reason why the similar effective behaviors are observed in the off-centered and the centered cases for the shear deformation, as shown in Fig. 2 in the manuscript.

For the y-directional dilatational deformation, the lumped stiffness models are described in Fig. S6. The overall effective stiffness (k_{eff}) in the case can be calculated by the parallel combination of lumped local stiffnesses k_{A} (left region), k_{B} (center), and k_{C} (right) unlike those by the series combinations in the previous x-dilatational and shear cases. The local stiffness in the regions above or below the slits may be negligible compared with that in the non-slit regions so that we can assume $k_{N,\text{offcen}} \approx k_{N1,\text{offcen}} \approx k_{N,\text{cen}} \approx k_{N1,\text{cen}}$ for $N = \text{A, C}$. The local stiffnesses for the center regions (k_{B}) are approximately the same in the present off-centered and centered cases. Consequently, we can estimate that $k_{\text{eff,offcen}} \approx k_{\text{eff,cen}}$, here. Figure

S6c obtained by numerical simulations confirms that the ratio $k_{\text{eff,offcen}}$ to $k_{\text{eff,cen}}$ is almost 1 regardless of h. In the y-directional dilatational case, therefore, the slit location as well as the slit length h has little influence on the overall effective stiffness because weak local stiffnesses in the neighborhood of the slits can be neglected in the parallel combinations of local stiffnesses.

Supplementary E. Validation of retrieved effective material parameters for the metamaterial

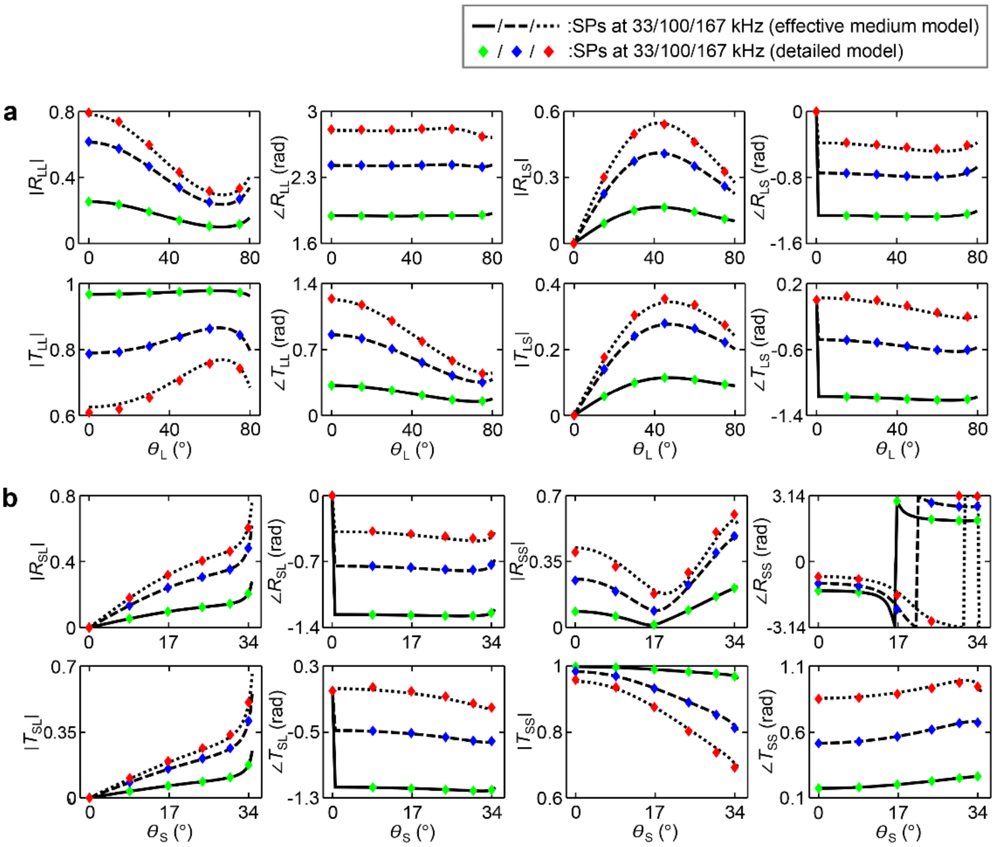

Figure S7. Scattering parameters of a single-period metamaterial slab shown in Fig. 3c at $f = 33$, 100, and 167 kHz for the varying wave incident angles of (a) $0^{\circ} \le \theta_{\mathrm{L}} \le 80^{\circ}$ for a longitudinal wave incidence and (b) $0^{\circ} \le \theta_{\mathrm{S}} \le 34^{\circ}$ for a shear wave incidence. Actual scattering parameters are in green, blue, and red diamonds while estimated effective material parameters are in black solid, dashed, and dotted lines, respectively.

In order to confirm the accuracy of the retrieved effective material parameters of the metamaterial shown in Fig. 3, we performed the validation by calculating the scattering parameters [2]. A single-period metamaterial slab with $h = 1.08$ mm (shown in Fig. 3c) is considered, here. In the scattering parameters, both the wavevector information and characteristic impedance information of the metamaterial are involved, which determine the full metamaterial behavior. The scattering parameters are calculated for the varying incident angles of $0^{\circ} \le \theta_{\mathrm{L}} \le 80^{\circ}$ (for longitudinal wave incidence) and $0^{\circ} \le \theta_{\mathrm{S}} \le 34^{\circ}$ (for shear wave incidence) in Figs. S7a and S7b, respectively, by using the retrieved effective material parameters at $f = 33$, 100, and 167 kHz (indicated with the black solid, dashed, and dotted

lines, respectively). These values are then compared to the actual ones (green, blue, and red diamonds, respectively). In the plots, the scattering parameters involved with reflection and transmission are defined as R_{IJ} and T_{IJ} where the first (I) and second (J) subscripts denote the modes of incident and scattered waves, respectively. Subscript L indicates a longitudinal wave mode while S indicates a shear wave mode. For instance, R_{SL} denotes the reflection coefficient of a longitudinal wave for a shear wave incidence.

From the results, one can find that the two sets of scattering parameters match well with each other for the entire range of incident angles considered. This observation confirms that the retrieved effective material parameters perfectly describe the actual behavior of the metamaterial.

Supplementary F. Simulation method for a tilted metamaterial

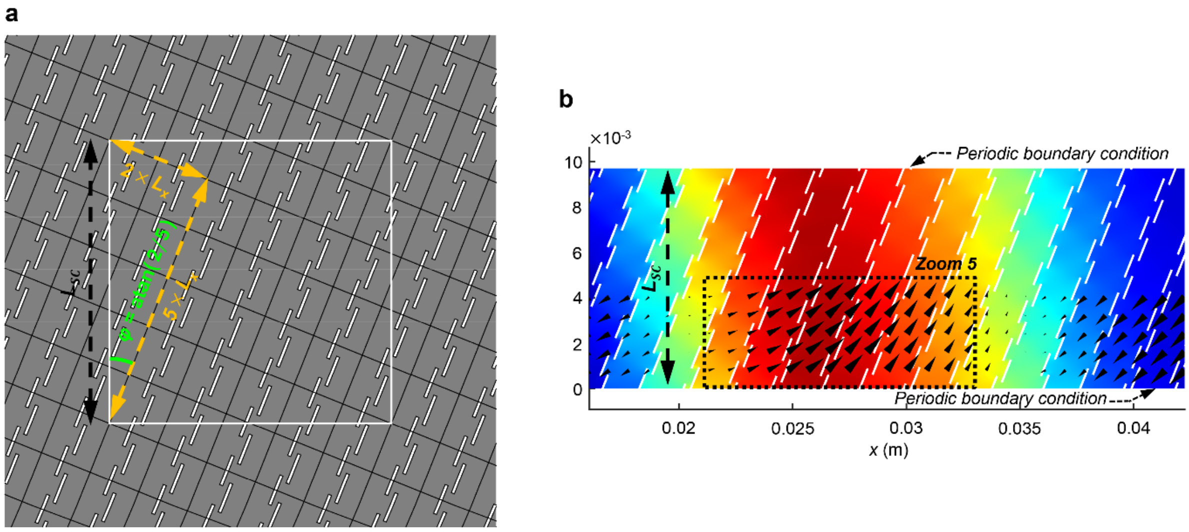

Figure S8. (a) Schematic configuration of the off-centered double-slit metamaterial that is tilted by $\varphi = \arctan(2/5)$. The square supercell block with the length of $L_{SC} = \sqrt{5^2 + 2^2} \times L$ is marked with the white solid lines. (b) The original simulation result of Fig. 5c in the manuscript, which is calculated by using the periodic boundary conditions on a pair of supercell edges: the "Zoom 5" region in Fig. 5c is denoted with black dotted lines.

Our scheme to impose the periodic boundary condition for the metamaterial consisting of tilted unit cells is to use a supercell. The metamaterial we wanted to work with in Fig. 5c consists of the rotated unit cell by $\varphi = 22^\circ$. Note that $\tan\varphi$ is nearly equal to $2/5$, i.e., $\varphi \approx \arctan(2/5) = 21.80^\circ$. When drawing a square with the length of $L_{SC} = \sqrt{5^2 + 2^2} \times L$ (L: lattice constant) in the geometry of the tilted metamaterial by $\varphi = \arctan(2/5)$ (see Fig. S8a), we can easily find that the block is a supercell. This argument indicates that the tilted metamaterial can be simulated by using the supercell with the periodic boundary conditions at the four edges. The simulation results shown in Fig. 5c in the manuscript was actually obtained from the supercell simulations, as shown in Fig. S8b (the "Zoom 5" region in Fig. 5c is marked with the black dotted lines). Because the wavefront inside the supercell is fairly flat in the macroscopic view, only the results for a half of the supercell region was presented in the manuscript.

Supplementary G. Group velocity characteristics of the metamaterial

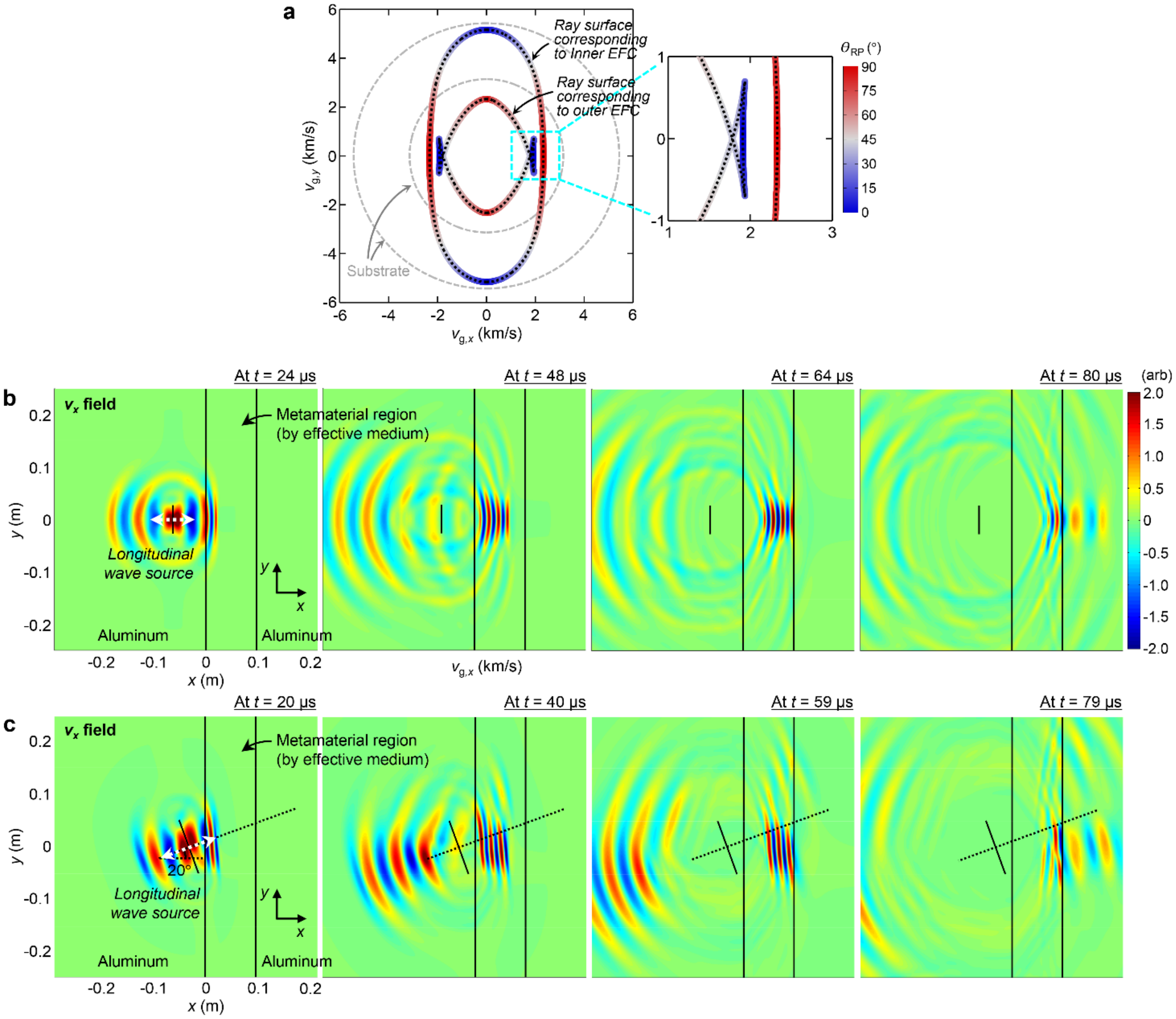

Figure S9. (a) The ray surface of the metamaterial with the slit length of $h = 1.08$ mm at $f = 100$ kHz is indicated by the black dotted lines with color that represents the relative polarization orientation θ_{RP} at the corresponding group velocity (v_{g}) vectors. The ray surface of aluminum substrate is also shown by the gray dashed lines. The triplication region is zoomed at the right. (b) The time-transient simulation results with the passage of time when a finite line source that generates a dominant longitudinal wave at the normal to the metamaterial slab is placed in front of the slab. (c) Similar simulations are conducted with a finite line source that generates a dominant longitudinal wave at the oblique angle of 20° with respect to the normal to the slab. In the simulations, the metamaterial slab is modeled by the effective medium.

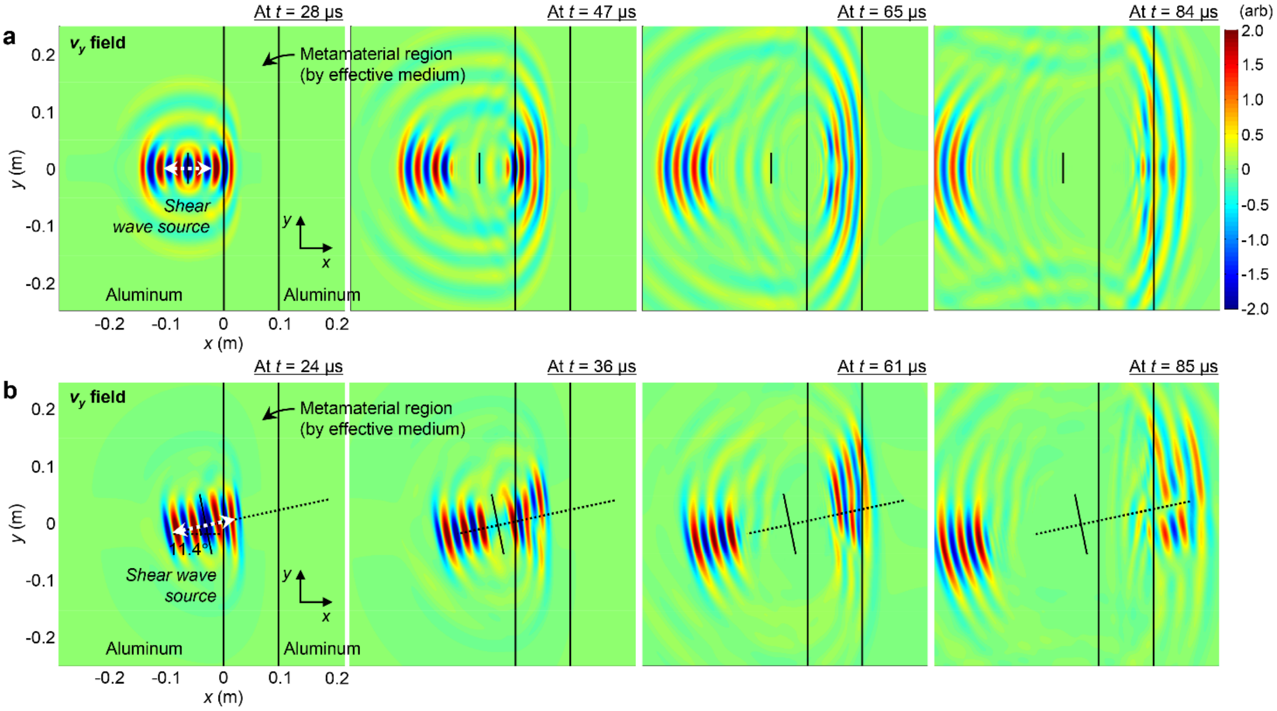

Figure S10. (a) Time-transient simulation results with the passage of time when a finite line source that generates a dominant shear wave at the normal to the metamaterial slab is placed in front of the slab. (b) Similar simulations are conducted with a finite line source that generates a dominant shear wave at the oblique angle of 11.4° with respect to the normal to the slab. In the simulations, the metamaterial slab is modeled by the effective medium.

Another extraordinary wave phenomenon that develops due to the polarization anomaly is the triplication [3-4] in a ray surface for a (quasi-)longitudinal mode. The triplication refers to a phenomenon that three ray surface components (i.e., group velocity vectors) share the same ray (group velocity) direction. The triplication phenomenon in a ray surface occurs only when the corresponding EFC profiles are locally concave. In the neighborhood of a triplication region, therefore, one can expect a wave focusing phenomenon. Note that only the solids exhibiting the polarization anomaly could have the triplications for a (quasi-)longitudinal mode because this phenomenon appears only in the ray surface corresponding to the outer (slow) EFC branch. In ordinary solids, the outer EFC branch corresponds to a (quasi-)shear mode. Due to the anomalous polarization transitions of the off-centered double-slit metamaterial, a (quasi-)longitudinal mode possibly lies in the outer branch so that the triplication phenomenon could be achieved for the (quasi-)longitudinal mode.

The ray surface of the proposed off-centered double-slit metamaterial with the slit length of $h = 1.08$ mm is calculated in Fig. S9a by using the retrieved effective material parameters at

$f = 100$ kHz. The ray surface is indicated by black dotted lines with the color representing the relative polarization angle (θ_{RP}) at each ray location. In the inner ray surface, which corresponds to the outer EFC branch, the triplications are clearly observed in the neighborhood of the *x*-axis. Due to the polarization anomaly of the metamaterial, the polarization in the triplication region is (quasi-)longitudinal, as unusual.

To examine the unusual triplication feature of the metamaterial, time-transient simulations were performed at $f = 100$ kHz in Fig. S9b,c: the finite length (L_{S}) of a longitudinal-wave line source is placed in front of the metamaterial slab (thickness of $9.64\ \mathrm{cm} = 1.77 \times \lambda_0^{\mathrm{L}}$ where λ_0^{L} is the longitudinal wavelength in aluminum), which is sandwiched by aluminum media. Here, the metamaterial slab is modeled by the effective medium for fast computation. In Fig. S9b, a line source with the length of $L_{\mathrm{S}} = \lambda_0^{\mathrm{L}}$ is used to generate a dominant longitudinal wave at the normal to the metamaterial slab. The *x*-directional velocity fields (v_x) are calculated with the passage of time. One can observe that the generated longitudinal wave is focused inside the metamaterial slab. After the wave passes through the slab, the wave is focused again. The results imply that the metamaterial indeed exhibits the triplication behavior for the (quasi-)longitudinal mode, which leads to the focusing of the wave mode. Note that the wave source used here generates non-zero k_y wave components since the source length is even comparable to the wavelength. To show the unique triplication behavior better, a line source with $L_{\mathrm{S}} = 2\lambda_0^{\mathrm{L}}$ is modeled to generate a dominant longitudinal wave at the oblique angle of 20° with respect to the normal to the slab (Fig. S9c). In the results, we can observe that the wave is negatively refracted at the first and the second slab boundaries.

In Fig. S10, the similar time-transient simulations were performed for a (quasi-)shear wave mode: a shear-wave line source with $L_{\mathrm{S}} = \lambda_0^{\mathrm{L}}$ for normal incidence is considered in Fig. S10a while a source with $L_{\mathrm{S}} = 2\lambda_0^{\mathrm{L}}$ for oblique incidence at 11.4° with respect to the normal to the slab is considered in Fig. S10b. From the results in Fig. S10a, one can observe that the generated shear wave undergoes significant diffraction inside the metamaterial slab. The wave phenomenon follows the ray analysis as described in Fig. S9a: the outer ray surface for a quasi-shear mode covers a wide range of v_{g} in the *y*-direction. The group velocity feature of a quasi-shear mode also can be seen in Fig. S10b where the wave is significantly refracted outward at the slab boundary. The proposed metamaterial is found to possibly diffract a quasi-shear mode and focus a quasi-longitudinal mode, unlike other ordinary solids.

Supplementary H. Robustness of the proposed wave mode converter

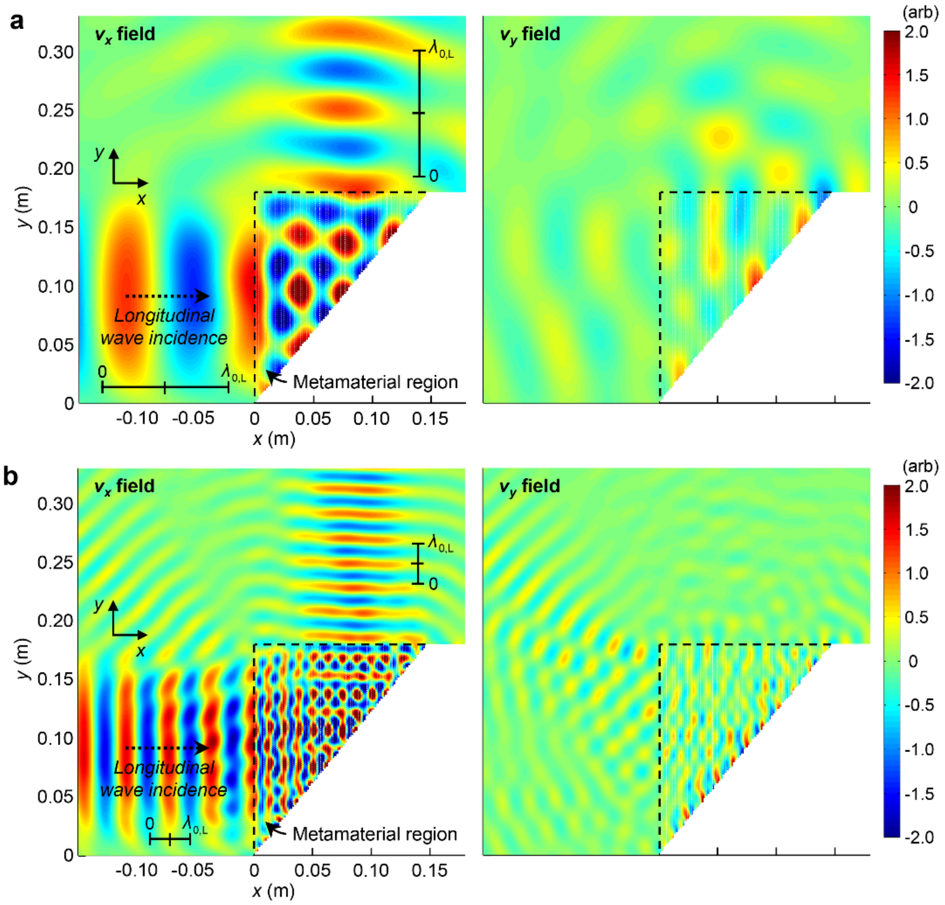

Figure S11. Time-harmonic simulations for the validation of the proposed wave mode converter at (a) $f = 50$ kHz and (b) 150 kHz. The x- (left) and y-directional (right) velocity (v_x or v_y) fields are presented. In the plots, the longitudinal wavelength λ_0^{L} in aluminum is indicated with a scale bar. The interfacing boundary between the converter and aluminum is marked with the black dashed lines.

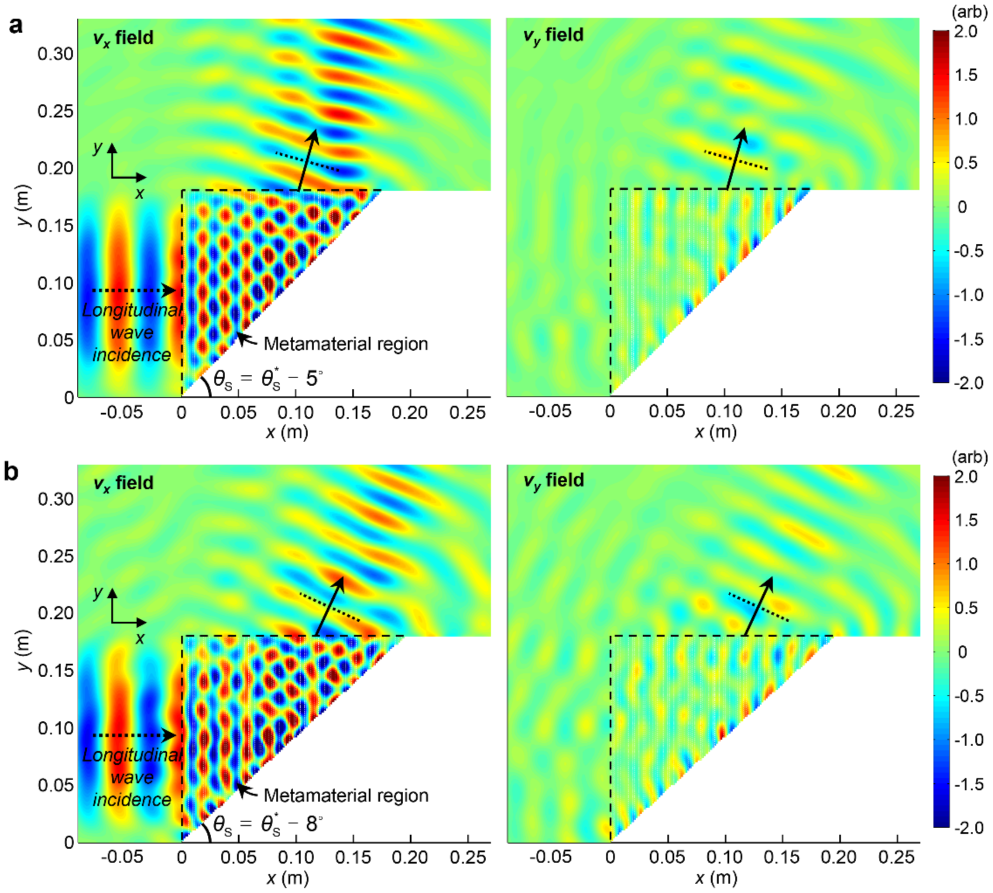

Figure S12. Time-harmonic simulations for the validation of the proposed wave mode converter at $f = 100$ kHz when the slope angle is changed to (a) $\theta_S = \theta_S^* - 5^\circ$ and (b) $\theta_S = \theta_S^* - 8^\circ$ where θ_S^* is the original slope angle. The results are represented by the x- (left) and y-directional (right) velocity (v_x or v_y) fields. The directions of the transmitted (quasi-)shear waves estimated from the theoretical calculation are illustrated by the black arrows. The interfacing boundary between the converter and aluminum is marked with the black dashed lines.

In order to examine if the proposed wave mode converter operates robustly over a broadband frequency range, we performed the same simulations with those in Fig. 6b but at other frequencies like at $f = 50$ kHz (Fig. S11a) and 150 kHz (Fig. S11b). The results are represented by the x- (left) and y-directional (right) velocity fields. For convenience, the longitudinal wavelength λ_0^L in aluminum, the surrounding material of the converter, is indicated with a scale bar. Regardless of frequency, the proposed converter virtually emits only a shear wave for the input of a longitudinal wave. The emitted wave virtually exhibits only the x-directional particle motions with negligible y-directional motion, confirming that the wave

mode is virtually shear. The observation also can be confirmed by the fact that the output wave has a shorter wavelength compared with that of the incident longitudinal wave.

Remarkably, the wave mode converting performance is well retained even at the low frequency for which the longitudinal wavelength is rather comparable with the dimension of the converter. At the high frequency around $f = 150$ kHz (Fig. S11b), there exists an unexpected wave scattering at the incline of the converter. (See the scattered wave in the left region of the converter that propagates at about 45° with respect to the left boundary of the converter.) It is due to the discretization of the incline in a zig-zag pattern, which cannot be considered to be sufficiently linear for waves of short wavelengths. By reducing the dimension of the constituent metamaterial unit cell further, we can avoid the scattering problem and thus achieve better mode conversion at high frequencies.

Other simulations are conducted to investigate if the proposed mode converter can be applied to the emission of shear waves at the oblique angle with respect to the converter boundary. For the validation, only the slope angle of the converter needs to be changed to $\theta_S = \theta_S^* - 5^\circ$ (Fig. S12a) and $\theta_S = \theta_S^* - 8^\circ$ (Fig. S12b) from the original angle (θ_S^*) considered in Fig. 6. The other simulation setups were unchanged and the selected frequency is 100 kHz. In each case, the transmission direction specifically for a shear wave is theoretically calculated by using the effective material parameters as illustrated with the black arrows in the plots. (For a longitudinal wave, the transmitted angle should be distinctively larger than that for a shear wave, although it is not shown here).

In the results, one can find that a single wave mode is transmitted from the converter and that the transmission angle follows the theoretical one. The wave exhibits dominant x-directional velocity fields but the y-directional fields also appear because the propagation direction is tilted from the principal axes. The observations confirm that the transmitted wave from the converter is dominantly shear. The wave mode converting performance is still retained even for the varying slope angles. Through adjusting the slope angle in an appropriate range, we can tailor the transmission direction of a converted shear wave, not only for the normal, but also for the oblique directions. In the engineering point of view, we believe that the robust performance of the converter would expand its potential to various applications.

Supplementary I. Re-design of the metamaterial unit cell for the fabrication

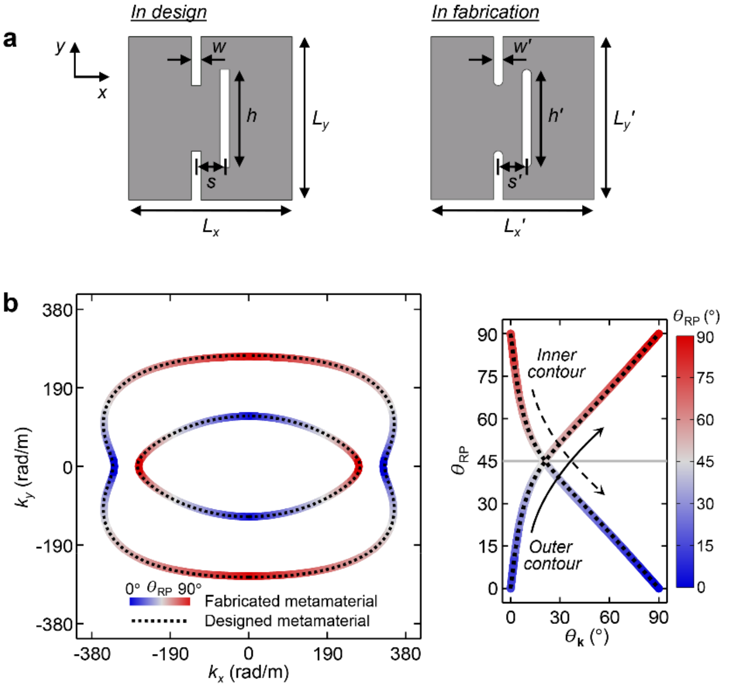

Figure S13. (a) Schematic configuration of the metamaterial unit cell considered in the design (left) and that considered in the fabrication (right). The geometric parameters for the former are indicated with (w, h, s, L_x, L_y) while those for the latter are with (w', h', s', L_x', L_y'). (b) The comparison of the EFCs (left) and the relative polarization orientation, θ_{RP} (right), between the fabricated (solid lines with color) and the designed (black dotted lines) versions.

In the fabrication of the present metamaterial, we cannot avoid the occurrence of fillets at the slit corners originating from the micro-machining errors. In order to achieve a similar effective behavior with that of the original configuration (shown in the left in Fig. S13a) in the fabrication, we slightly modified the unit cell configuration after taking the fillets into account (shown in the right). The re-designed geometric parameters are as follows: $L_x' = 1.8$ mm, $L_y' = 1.8$ mm, $w' = 0.11$ mm, $h' = 1.10$ mm, and $s' = 0.3$ mm.

In order to investigate if the targeted effective behavior is achieved with the fabricated configuration, we calculated the EFCs (left) and the relative polarization orientation, θ_{RP} (right) at 100 kHz in Fig. S13b. The results (indicated with the solid lines with color) are compared with those of the original configuration (black dotted lines). As seen in the results, the two sets of results are virtually the same, confirming that the accurate realization of the targeted metamaterial behavior in the fabrication. The modified geometric parameters, w', h',

s', L_x', and L_y', are used to fabricate the metamaterial-based wave mode converter shown in Fig. 7.

Supplementary J. Meander-type magnetostrictive transducers

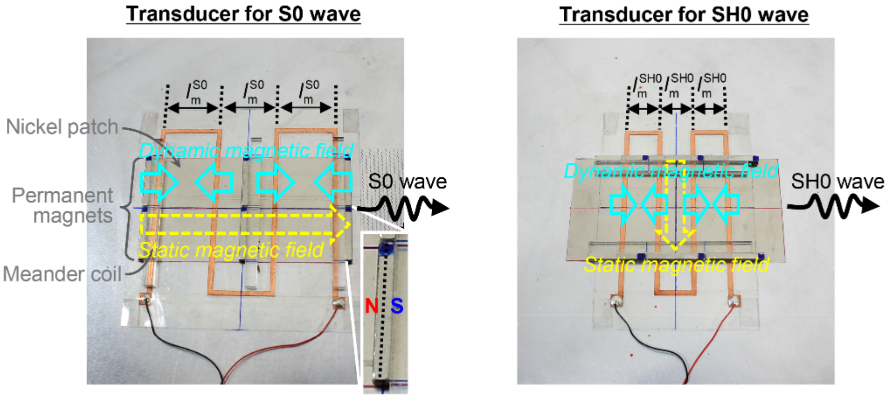

Figure S14. Meander-type magnetostrictive transducers are used for the generation and measurement of the lowest symmetric Lamb wave (left) and the lowest shear-horizontal wave (right) in a thin plate. The transducers consist of a nickel patch, permanent magnets, and a meander coil.

The meander-type magnetostrictive transducers were employed in the experiments (in Fig. 7) to generate and measure the lowest symmetric Lamb wave, namely the S0 wave, and the lowest shear-horizontal wave, namely the SH0 wave, in a thin plate. The transducers consist of a nickel patch, permanent magnets, and a meander coil, which operate in accordance with the principle of magnetostriction. The coupling phenomenon between magnetic field and mechanical strain occur in ferromagnetic materials such as nickel. If bias static magnetic field by permanents magnets and dynamic magnetic field by meander coil are simultaneously applied in the same direction to a nickel patch (see the S0 wave case in Fig. S14), the patch is supposed to have dominant longitudinal deformation in the magnetic field direction. Because the meander coil itself has a role as a wavelength filter, one can generate virtually a single wave mode by selecting the intervals between the adjacent meander coil lines (l_{m}) to match with half the wavelength of a targeted wave. For generation of the S0 wave at $f = 100\ \mathrm{kHz}$, the coil line intervals are set as $l_{\mathrm{m}} = l_{\mathrm{m}}^{\mathrm{S0}} = 2.7\ \mathrm{cm} \approx \lambda_0^{\mathrm{S0}}/2$ where λ_0^{S0} is the wavelength of the S0 wave in an aluminum plate at 100 kHz. In the transducers for the SH wave, meanwhile, the direction of bias static magnetic field by permanents magnets is changed to be orthogonal to the direction of the dynamic magnetic field by a meander coil (see the SH0 wave case in Fig. S14). Under this setup, the nickel patch has dominant shear deformation. To achieve high selectivity of the SH0 wave at $f = 100\ \mathrm{kHz}$, the coil line intervals are set as $l_{\mathrm{m}} = l_{\mathrm{m}}^{\mathrm{SH0}} = 1.6\ \mathrm{cm} \approx \lambda_0^{\mathrm{SH0}}/2$ where λ_0^{SH0} is the wavelength of SH0 wave in an aluminum plate at 100 kHz. The transducers can be also used to measure the S0 and SH0 waves, respectively, in accordance with the reversed magnetostrictive phenomenon. The detailed mechanisms of the magnetostrictive

transducers and their performances can be found in the previous studies [5-6].

Supplementary K. Calibration procedure for S0 and SH0 transducers

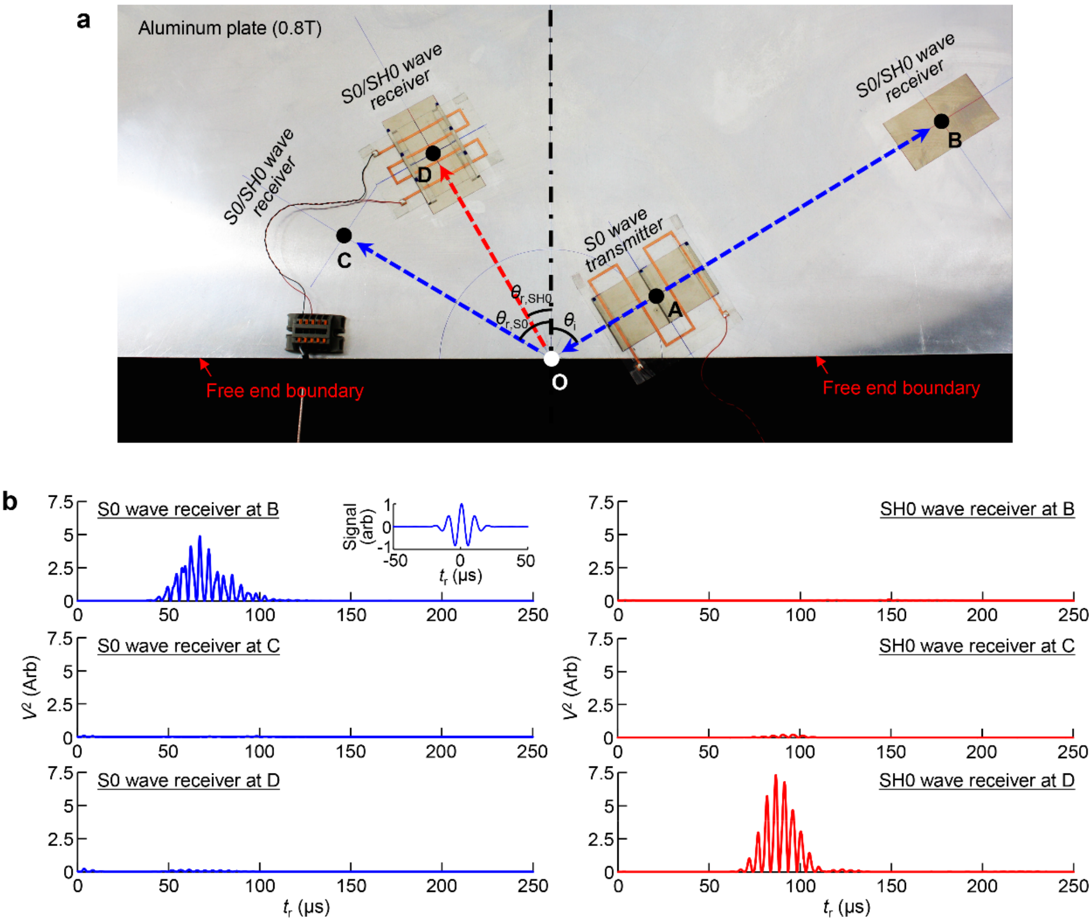

Figure S15. (a) Photo of the experimental setups to calibrate the transducers. The S0 wave is generated at location A in an aluminum plate. The reflected S0 and SH0 waves by the free-end boundary are measured at locations C and D, respectively. For reference, the generated S0 wave is also measured at location B. (b) The square of the measured voltage at locations B (first row), C (second row), and D (third row) are plotted when the receivers for the S0 (left) and SH0 (right) waves are used. As an input signal, a modulated-Gaussian pulse with the center frequency of $f = 100$ kHz is used (inset).

In order to calibrate the used magnetostrictive transducers for S0 and SH0 waves, an additional experiment was conducted with experimental setups shown in Fig. S15a. At location A, which is 10-cm away from location O, the S0 wave is generated by the S0-wave transducer towards the free-end boundary in an aluminum plate with the incident angle θ_i. As an input signal, a modulated Gaussian pulse with the center frequency of $f = 100$ kHz is used. The reflected S0 and SH0 waves at the angles of $\theta_{r,S0}$ and $\theta_{r,SH0}$ are measured at locations C and D, respectively, which are both 20-cm away from location O. Note that the reflection angles were

predicted in theory. For reference, the generated S0 wave at location A is also measured at location B by using the S0- and SH0-wave transducers, respectively, where location B is 30-cm away from location A.

In Fig. S15b, the measured signals that are represented by the square of voltage (V^2) at locations B (first row), C (second row), and D (third row) by using the transducers for the S0 (left) and SH0 (right) waves are presented. The measured voltage by the present magnetostrictive transducers is directly related to strain so that the square of voltage can be represent strain energy. In theory, if the incident angle is set as $\theta_i = 59.4^\circ$, no reflection occurs for the S0 wave and the reflection totally involves the SH0 wave. In the setups, therefore, the strain energy of the S0 wave measured at location B is supposed to be approximately the same as that of SH0 wave measured at location D (according to the energy conservation law). The above theoretical predictions are well found in the present experimental results (see the negligible measured strain energy for the S0 and SH0 waves at location C and the dominant strain energy for the S0/SH0 wave at location B/D). From the measured strain energy amplitudes of the S0 and SH0 waves at locations B and D, respectively, we successfully calculated the sensitivities of the transducer for measuring each wave mode. The experimental results shown in Fig. 7 were derived by using the sensitivities.

Supplementary L. Fabrication error

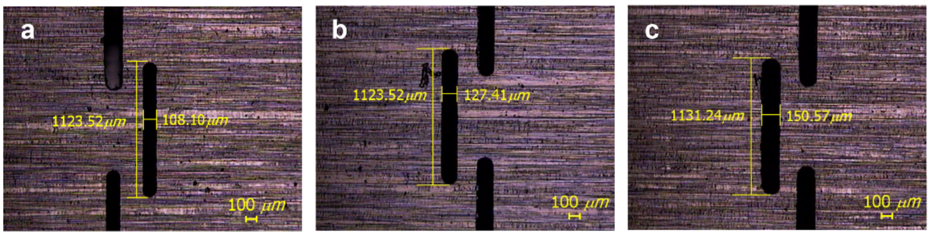

Figure S16. Zoomed views on the fabricated unit cells in the elastic wave mode converter shown in Fig. 7a. The unit cell examples have (a) small, (b) medium, and (c) large machining errors.

Due to the abrasive fabrication tools during micro-machining, some fabrication errors cannot be avoided. In Fig. S16, for example, fabricated metamaterial unit cell images in the elastic wave mode converter, as shown in Fig. 7a, are prepared. Considering the original configuration in the design, some fabrication errors were found especially in the geometric parameters of slit width (w) and length (h). In the original design, we had $w = 0.110$ mm and $h = 1.100$ mm. In the simulations shown in Fig. 7d, the fabrication errors on slit width (w) and length (h) were considered with small and large errors in Simulations 2 and 3, respectively.

References

1. Helbig, K. & Schoenberg, M. Anomalous polarization of elastic waves in transversely isotropic media. *J. Acoust. Soc. Am.* **81**, 1235-1245 (1987).

2. Lee, H. J., Lee, H. S., Ma, P. S. & Kim, Y. Y. Effective material parameter retrieval of anisotropic elastic metamaterials with inherent nonlocality. *J. Appl. Phys.* **120**, 104902 (2016).

3. Vavryčuk, V. Generation of triplications in transversely isotropic media. *Phys. Rev. B* **68**, 054107 (2003).

4. Vavryčuk, V. Calculation of the slowness vector from the ray vector in anisotropic media. *P. Roy. Soc. Lond. A Mat.* **462**, 883-896 (2006).

5. Kim, H. W., Kwon, Y. E., Cho, S. H. & Kim, Y. Y. Shear-horizontal wave-based pipe damage inspection by arrays of segmented magnetostrictive patches. *IEEE T. Ultrason. Ferr.* **58**, 2689-2698 (2011).

6. Kim, Y. Y. & Kwon, Y. E. Review of magnetostrictive patch transducers and applications in ultrasonic nondestructive testing of waveguides. *Ultrasonics* **62**, 3-19 (2015).
